# Uncertainty quantification of parenchymal tracer distribution using random diffusion and convective velocity fields

**DOI:** 10.1186/s12987-019-0152-7

**Published:** 2019-09-30

**Authors:** Matteo Croci, Vegard Vinje, Marie E. Rognes

**Affiliations:** 1 Mathematical Institute, University of Oxford, Oxford, UK; 2 Department for Numerical Analysis and Scientific Computing, Simula Research Laboratory, P.O.Box 134, 1325 Lysaker, Norway

**Keywords:** Cerebrospinal fluid, Interstitial fluid, Diffusion, Convection, Glymphatic system, Paravascular space, Uncertainty quantification

## Abstract

**Background:**

Influx and clearance of substances in the brain parenchyma occur by a combination of diffusion and convection, but the relative importance of these mechanisms is unclear. Accurate modeling of tracer distributions in the brain relies on parameters that are partially unknown and with literature values varying by several orders of magnitude. In this work, we rigorously quantified the variability of tracer distribution in the brain resulting from uncertainty in diffusion and convection model parameters.

**Methods:**

Using the convection–diffusion–reaction equation, we simulated tracer distribution in the brain parenchyma after intrathecal injection. Several models were tested to assess the uncertainty both in type of diffusion and velocity fields and also the importance of their magnitude. Our results were compared with experimental MRI results of tracer enhancement.

**Results:**

In models of pure diffusion, the expected amount of tracer in the gray matter reached peak value after 15 h, while the white matter did not reach peak within 24 h with high likelihood. Models of the glymphatic system were similar qualitatively to the models of pure diffusion with respect to expected time to peak but displayed less variability. However, the expected time to peak was reduced to 11 h when an additional directionality was prescribed for the glymphatic circulation. In a model including drainage directly from the brain parenchyma, time to peak occured after 6–8 h for the gray matter.

**Conclusion:**

Even when uncertainties are taken into account, we find that diffusion alone is not sufficient to explain transport of tracer deep into the white matter as seen in experimental data. A glymphatic velocity field may increase transport if a large-scale directional structure is included in the glymphatic circulation.

## Introduction

Over the last decade, there has been a significant renewed interest in the waterscape of the brain; that is, the physiological mechanisms governing cerebrospinal fluid (CSF) and interstitial fluid (ISF) flow in (and around) the brain parenchyma. A number of new theories have emerged including the glymphatic system [1, 2], the intramural periarterial drainage (IPAD) theory [3, 4], and the Bulat–Klarica–Oreskovic hypothesis [5], along with critical evaluations [6–9]. A great deal of uncertainty and a number of open questions relating to the roles of diffusion, convection and clearance within the brain parenchyma remain.

Exchange between CSF and ISF is hypothesized to occur along small fluid-filled spaces surrounding large penetrating arteries in the brain parenchyma known as paravascular spaces (PVS) [1, 10]. Tracer has been observed to move faster in paravascular spaces in response to increased arterial pulsations, and arterial pulsation has thus been proposed as the main driver of paraarterial flow [11–13]. After entering the extracellular space (ECS), a bulk flow of ISF from paraarterial to the paravenous spaces has been proposed to occur before re-entry to the the subarachnoid space (SAS) [2]. This concept of CSF/ISF fluid circulation has been named the glymphatic system, with bulk flow as a mechanism for effective waste clearance from the brain parenchyma. Xie et al. [14] showed glymphatic influx to increase in sleeping mice, linking the importance of sleep to clearance of waste products. Sleep was also associated with an increased interstitial space volume fraction, a possible explanation for increased flow through the interstitial space. MRI investigations have also found evidence for glymphatic function in human brains [15, 16].

While several studies demonstrate CSF influx along paraarterial spaces [1, 13, 17, 18], the efflux route is more debated. Carare et al. [3] found evidence of solutes draining from the brain parenchyma along basement membranes of capillaries and arteries, going in the opposite direction of blood flow and possible PVS fluid movement. This flow is however not facilitated by arterial pulsations [19], but by the movement of smooth muscle cells [20]. Bedussi et al. [21] observed tracers move towards the ventricular system, ultimately leaving the brain via the cribriform plate and the nose. A continuous pathway alongside capillaries to the paravenous space has been suggested [22], and capillaries continuously filtrate and absorb water inside the brain parenchyma [5, 6], although not necessarily with a net flux of water [23]. In addition, substances may leave the parenchyma crossing the blood-brain barrier, or possibly directly to lymph nodes [24].

In a recent review, Abbott and colleagues [25] concluded that bulk flow within the parenchyma is likely to be restricted to the PVS and possibly white matter tracts. Earlier studies have reported a bulk flow velocity magnitude of less than 1  µm/s [26], while recent evidence suggests average net bulk flow of around 20 µm/s, restricted to the PVS [13, 27]. Nevertheless, since tracer movement in in-vivo studies does not necessarily directly reflect underlying fluid flow [28], the exact velocity field governing ISF flow in the brain remains unknown.

All of the aforementioned in-vivo studies have used tracers or micro-spheres to track the movement of fluid within the intracranial space. Injection of fluid at rates as low as 1 µL/min can cause a significant increase of local intracranial pressure (ICP) [29], which may lead to pressure gradients driving bulk flow. On the other hand, non-invasive methods such as diffusion tensor imaging may serve as a promising tool due to its sensitivity to dispersion and bulk flow. This method has been applied successfully to demonstrate increased diffusivity with vascular pulsation compared to diastole [30]. The diffusion coefficient was found to be anisotropic and highest parallel to PVS, however a value of the bulk fluid velocity magnitude could not be reported from these measurements. In addition to both invasive and non-invasive experiments, computational models have been used to assess the possibility and plausibility of bulk flow within the parenchyma. Tracer movement in the extracellular space has been found to be dominated by diffusion [31], a conclusion similar to that of Smith et al. [9] in experimental studies with very low infusion rates.

Even though computational models can distinguish between diffusion and bulk flow, a major challenge remains with regard to the unknown material parameters, boundary conditions and other model configurations needed to accurately predict the movement of ISF in the brain parenchyma. For instance, the permeability of brain tissue used in computational models varies from $10^{-10}$ to $10^{-17}\,\hbox {m}^2$ [31, 32]. Because the permeability is directly linked to the Darcy fluid velocity in these models, this parameter choice could result in a difference of 7 orders of magnitude in predicted ISF flow. In addition, CSF dynamics vary between subjects [33] and human CSF production has been reported to increase in the sleeping state [34] which may alter ISF flow. Recently it has been pointed out that there is an overarching need to reduce uncertainty when characterizing the anatomy and fluid dynamics parameters in models considering the glymphatic circulation [35].

Replacing partial differential equation (PDE) parameters subject to uncertainty with spatially correlated random fields is a common modelling choice in the uncertainty quantification (UQ) literature [36–38] and Monte Carlo methods have been successfully used in biology to quantify how uncertainty in model input propagates to uncertainty in model output. However, these methods have mainly been applied to simulations of the cardiovascular system [39, 40] and, to our knowledge, there has only been one study in which Monte Carlo methods have been used for UQ in brain modelling [41]. To the authors’ knowledge, there has been no previous work on forward uncertainty quantification for simulations of tracer transport with the brain parenchyma.

### Study outline

With this study, we aim to rigorously quantify how the aforementioned uncertainties in the physiological parameters and in ISF flow affect the spread of a tracer from the SAS into the brain parenchyma. We assume movement of tracer in the brain parenchyma to occur by diffusion and/or convection. To account for uncertainty and variability, we circumvent the lack of precise parameter values by modelling velocity and diffusivity as stochastic (random) fields. We then set up a stochastic^1^ PDE model with these random fields as coefficients and quantify the uncertainty in the model prediction via the Monte Carlo (MC) method.

More specifically, we model the MRI study performed by Ringstad et al. [15], assessing glymphatic function in the human brain, and derive a baseline convection–diffusion–reaction PDE. The model coefficients are designed to represent different hypotheses on CSF flow and clearance, including diffusion, the glymphatic system and possible capillary absorption, and uncertainty within each hypothesis. A total of five different models were investigated, each with stochastic model coefficients. For each model, we compute the expected values and $99.73\%$ prediction intervals for different quantities of interest. The results reported in the study by Ringstad et al. are compared with the range of uncertainty in our model. We find that although the uncertainty associated with diffusion yields great variability in tracer distribution, diffusion alone is not sufficient to explain transport of tracer deep into the white matter as seen in experimental data. A glymphatic velocity field may increase tracer enhancement, but only when adding a large-scale directional structure to the glymphatic circulation.

## Methods

### In vivo evidence of tracer distribution to the brain

We model the MRI-study of Ringstad et al. [15]. In their experiments, 0.5 mL of 1.0 mmol/mL of the contrast agent gadobutrol was injected intrathecally, and used as CSF tracer in 15 hydrocephalus patients and eight reference subjects. The localization of the tracer was found with MRI at 4 different time periods, at 1, 3, 4.5, and 24 h following the injection. After 3 h, tracer was localized in the ventral region of the cranial SAS, and had started to penetrate into the brain parenchyma of the reference subjects. The following day it had spread throughout the brain tissue. Tracer was found to penetrate along large surface arteries in all study subjects, and a low proportion of tracer was found at the dorsal regions of the brain.

### Mathematical model for tracer movement in the brain parenchyma

We consider the following time-dependent partial differential equation to model transport of tracer in the brain parenchyma: find the tracer concentration *c* such that

1 $$\begin{aligned} \dot{c} + \nabla \cdot (v c ) - \nabla \cdot (D^{*} {{\,\mathrm{\nabla }\,}}c) + r c= 0. \end{aligned}$$

This Eq. (1) is assumed to hold for all times $t> 0$ and for all points in a spatial domain $\mathcal {D}$. The superimposed dot represents the time derivative, $D^{*}$ is the effective diffusion coefficient of the tracer in the tissue (depending on the tracer free diffusion coefficient and the tissue tortuosity) [26], *v* represents a convective fluid velocity and $r \ge 0$ is a drainage coefficient potentially representing e.g. capillary absorption [5] or direct outflow to lymph nodes [15]. We assume that the parenchymal domain contains no tracer initially: $c = 0$ at time $t = 0$.

This model thus requires as input two key physical parameters: the bulk fluid velocity *v* and gadobutrol diffusivity $D^{*}$ everywhere within the parenchyma. To investigate and compare different hypotheses for parenchymal ISF flow and tracer transport under uncertainty, we consider 5 stochastic model variations of Eq. (1). We consider two models with a stochastic diffusion coefficient (Models D1 and D2), and three models with stochastic velocity fields (Models V1, V2, and V3). Models D1 and D2 assume a negligible fluid velocity in the parenchyma ($v = 0$) and ignore capillary absorption or other direct outflow pathways ($r = 0$). For the velocity models (V1, V2 and V3), we consider a non-stochastic diffusion coefficient in order to isolate the effects of the stochastic velocity fields. An overview of the models is presented in Table 1.

**Table 1:** Summary of stochastic model variations with effective diffusion coefficient $D^{*}$, convective fluid velocity *v*, and drainage coefficient *r* in (1)

Model	$D^{*}$	*v*	*r*
D1	Random variable	0	0
D2	Random field	0	0
V1	Constant	Random influx and outflux field	0
V2	Constant	Model V1 + additional velocity field	0
V3	Constant	Random influx field	$r > 0$

#### Domain and geometry

**Fig. 1:**
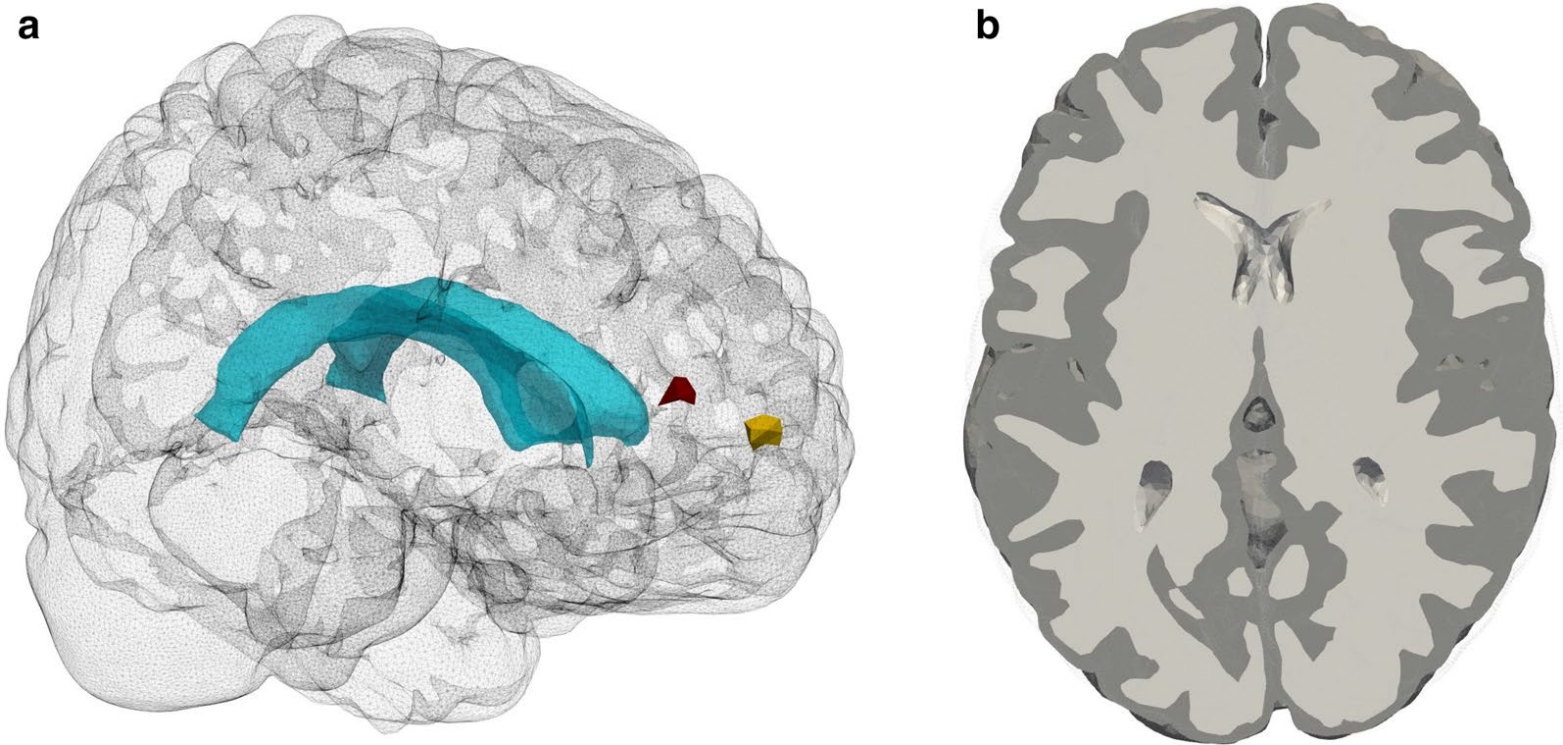
Computational domain. **a** The computational domain representing the brain parenchyma including the cerebellum. The interior lateral ventricles are marked (light blue) in the central region of the domain. Two smaller regions of interest $S_g$ and $S_w$, in the gray and white matter respectively, are marked in red ($S_w$) and yellow ($S_g$). **b** Representation of the gray (darker gray) and white matter (lighter gray) in the computational domain (axial slice)

We define the computational domain $\mathcal {D}$ as the union of white and gray matter from the generic Colin27 human adult brain atlas FEM mesh [42] version 2 (Fig. 1). This domain includes the cerebellum. The levels of the foramen magnum, the Sylvian fissure and the precentral sulcus are well represented by z-coordinates − 0.1, 0 and 0.1 m, respectively. The plane z = 0 corresponds approximately to the level of the lateral ventricles.

#### Boundary conditions modelling tracer movement in the SAS

Let $\partial D$ be the boundary of $\mathcal {D}$ and let $\partial \mathcal {D} = \partial \mathcal {D}_S \cup \partial \mathcal {D}_V$, with $\partial \mathcal {D}_S$ representing the interface between the brain parenchyma and the SAS, and $\partial \mathcal {D}_V$ representing the interface between the brain parenchyma and cerebral ventricles, respectively. We consider the following boundary conditions for (1):

2 $$\begin{aligned} c = g(c) \text { on } \partial \mathcal {D}_S, \end{aligned}$$

3 $$\begin{aligned} D^{*}{{\,\mathrm{\nabla }\,}}c \cdot n = 0 \text { on } \partial \mathcal {D}_V. \end{aligned}$$

In particular, we assume that a tracer concentration is given at the SAS interface (2) and no ventricular outflux (3). The dependence of *g* on *c* in (2) is detailed below. For clarity in presentation, we here first write *c* as a field depending on space and time only (and not on random events).

The boundary condition (2) models the movement of tracer starting from the lower cranial SAS and traveling upward in the CSF surrounding the brain as observed in the study by Ringstad et al. [15]. In particular, we let

4 $$\begin{aligned} \begin{aligned} g(c)(t, x)&= c_{\mathrm{CSF}}(t) \, h(t, x), \\ h(t, x)&= \left( 0.5+\frac{1}{\pi }\arctan (-a(x_3 - z_0 - u_{x_3}t))\right) , \end{aligned} \end{aligned}$$

for $x = (x_1, x_2, x_3) \in \mathcal {D}$. Here, at time *t*, $c_{\mathrm{CSF}}(t)$ is the average tracer concentration in the SAS, while *h*(*t*, *x*) represents its spatial distribution.

The expression for *h* is based on the following considerations. We assume that the diffusive and/or convective movement of tracer from the spinal to the cranial SAS over time is known, and we thus model *h*(*t*, *x*) as a smooth step function upwards (in the $x_3$-direction). In (4), $u_{x_3}$ represents the speed of tracer movement upwards in the SAS, and *a* reflects the gradient of tracer concentration from the lower to the upper cranial SAS. Finally, we assume that at time $t = 0$, the tracer has spread up to a relative distance of $z_0$ from the lateral ventricles. This specific expression for *h*(*t*, *x*) and the values of parameters *a*, $z_0$ and $u_{x_3}$ are based on the spread of tracer seen in the MR-images in the study by Ringstad et al. [15]. In particular, we use $a = 20\,\hbox {m}^{-1}$, $u_{x_3} = 1.5 \times 10^{-5}$ m/s and $z_0 = -0.2$ m. These parameters were chosen to match time to peak in three different regions in the CSF space in reference individuals [15].

To derive an expression for $c_{\mathrm{CSF}}$ in (4), we consider the conservation of tracer. We model the spread of $n_0 = 0.5$ mmol tracer in the CSF, assuming a volume of $V_\text {CSF} = 140$ mL CSF in the human SAS and ventricles [43]. The average concentration in the SAS right after injection is thus $c_{\mathrm{CSF}}(0)$ = 0.5 mmol/140 mL = 3.57 mol/$\hbox {m}^3$. At any given time, we assume that the total amount of tracer in the brain and in the SAS plus or minus the tracer absorbed or produced stays constant in time, and is equal to the initial amount $n_0 = 0.5$ mmol:

5 $$\begin{aligned} \int _\mathcal {D} c(t,x) \, \mathrm {d} x+ c_{\mathrm{CSF}}(t) V_{\mathrm{CSF}} + \int _0^t \int _\mathcal {D} r c(\tau ,x) \, \mathrm {d} x\, \mathrm {d} \tau = n_0 . \end{aligned}$$

By rearranging, we thus obtain an explicit expression for $c_{\mathrm{CSF}}$ that can be inserted into (4). It should be noted that the boundary concentration, as described by Eq. (4), depends on the tracer concentration in the brain parenchyma itself. Therefore, the boundary concentration will vary from simulation to simulation depending on how fast tracer spreads to the parenchyma.

### Modelling uncertainty via random variables and fields

A standard approach to model experimental variability or uncertainty in the input parameters is via stochastic modelling, and random variables or random fields in particular, see e.g. [44–46]. For clarity, we give a brief introduction to random variables and fields in this section. For further reading, we refer the reader to the books by Bland [47] or Jaynes [48]. We further detail the stochastic diffusion and velocity models in the subsequent sections.

We indicate a variable *X* whose value is subject to error or uncertainty (e.g. to reflect patient variability or uncertainty in its value) with $X(\omega )$, where $\omega$ is called an *event* and indicates a specific instance of $X(\omega )$, called a *sample* or a *realization*. Practically speaking, here $\omega$ can be seen as a given computer simulation. A quantity like *X* is called a *random variable* as its value $X(\omega )$ is not known a priori, but is fixed at each event (simulation) $\omega$. The values taken by a random variable are not arbitrary, but depend on the variable’s *probability density function* which reflects how likely each value is to happen, see e.g. [47] for further reading.

The average value attained by a random variable *X* is called its *expected value* or *expectation* and is indicated by $\mathbb {E}[X]$. Conversely, the variance of *X* is a measure of how much values $X(\omega )$ can differ from the average, i.e. the variability of $X(\omega )$ across events (simulations). The variance is indicated by $\mathbb {V}[X]$ and is given by $\mathbb {V}[X] = \mathbb {E}[(X-\mathbb {E}[X])^2]$. The expected value of a random variable can be approximated by taking the average across many samples of $X(\omega )$:

6 $$\begin{aligned} \mathbb {E}[X] \approx \frac{1}{N} \sum _{n=1}^N X(\omega ^n), \end{aligned}$$

where the sample size *N* is the number of realizations of $X(\omega )$ taken. The process of sampling $X(\omega )$ and of estimating $\mathbb {E}[X]$ by taking the sample average is the basis of the *Monte Carlo method* (see [49] and the references therein for further reading).

Random variables are constant i.e. do not vary in space. To represent spatially-varying functions (i.e. *fields*) with uncertain function values, we introduce *random fields*. A random field is a function of space whose value at each point *x* in the (three-dimensional) spatial domain $\mathcal {D}$ is given by a random variable. We write a random field *Y* as $Y(x, \omega )$ for spatial points *x* and events (simulations) $\omega$, to indicate that *Y* varies both across space and simulations. A sample or realization of the random field can then be viewed as a function of space $Y(\cdot , \omega )$. The expected value of a random field $\mathbb {E}[Y(x,\omega )] = \mu (x)$, where $\mu (x)$ is the mean function (which thus varies in space). The random variables that form the field are typically correlated among each other. This correlation is quantified by the *covariance function* *C*(*x*, *y*) that gives the covariance between $Y(x, \omega )$ and $Y(y, \omega )$ for two spatial locations *x* and *y* for each event $\omega$. Specifically, $C(x,y)= \mathbb {E}[(Y(x, \omega ) - \mu (x))(Y(y, \omega ) - \mu (y))]$.

In this study, we employ Matérn random fields [50] [see Additional file 1 (Section A) for more details] for modelling spatially varying parameters which are either unknown or subject to errors. Our choice is motivated by two primary reasons: first, Matérn fields are a standard choice for modelling random spatial variability in spatial statistics [51–53] and second, Matérn fields can be sampled much more efficiently than other Gaussian fields with general covariances [54]. A Matérn random field is characterized by its *correlation length* $\lambda$ which represents the distance past which point values of the field are approximately uncorrelated. Informally, this means that in each realization of the Matérn field, there are regions of length proportional to $\lambda$ within which the values of the field are similar.

In the following, we introduce stochastic representations of the effective diffusion coefficient $D^{*}$ and velocity *v*. We then write $D^{*}(\omega )$ when representing $D^{*}$ as a random variable, $D^{*}(x, \omega )$ when representing $D^{*}$ as a random field, and $v(x, \omega )$ when representing *v* as a random field. As a consequence, the tracer concentration solution of (1) thus depends on time, space and random events and can be expressed as $c = c(t, x, \omega )$.

### Stochastic diffusion modelling

The parenchymal effective diffusion coefficient of a solute, such as e.g. gadobutrol, is heterogeneous [55] (varies in space) and individual-specific (varies from individual to individual). Diffusion tensor imaging [56] provides evidence for such heterogeneity. To investigate the effect of uncertainty in the diffusion coefficient, we consider two approaches: first, to model the diffusion coefficient as a random variable and second, to model the diffusion coefficient as a random field, thus allowing for tissue heterogeneity. Both approaches are described in further detail below.

#### Effective diffusion coefficient modelled as a random variable

**Fig. 2:**
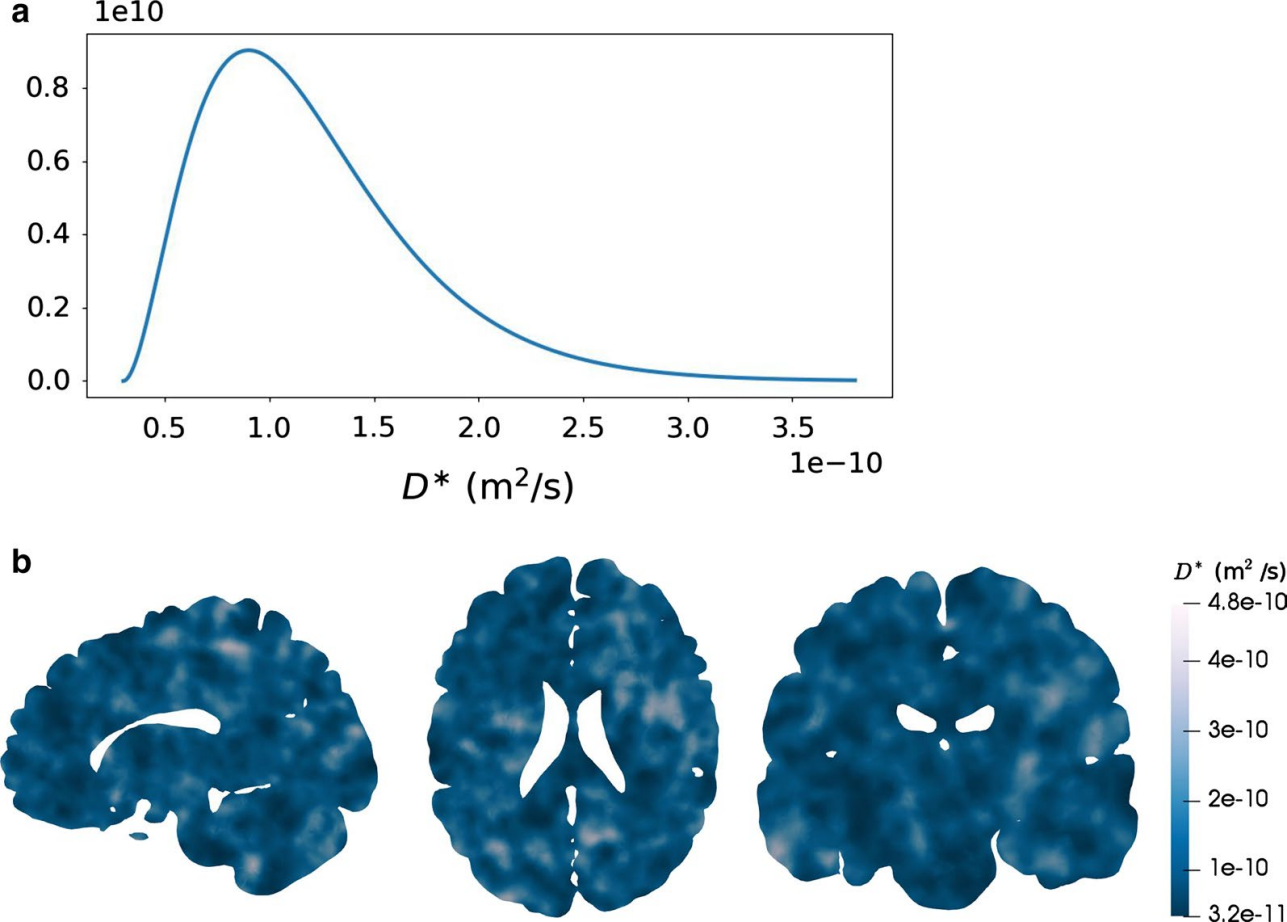
Stochastic diffusion coefficient models. **a** Assumed probability distribution of the homogeneous effective diffusion coefficient $D^{*}$ modelled as a random variable and used in Model D1. The expected value $E[D^{*}]$ is $1.2 \times 10^{-10}\,\hbox {m}^2$/s. **b** Sample of the heterogeneous effective diffusion coefficient (sagittal, axial and coronal slices ordered from left to right) modelled as a random field and used in Model D2

First, we consider the simplifying but common assumption that the effective diffusion coefficient is constant in space. We account for the uncertainty in its value by modelling it as a random variable depending on an event $\omega$:

7 $$\begin{aligned} D^{*}(\omega ) = 0.25 D^{*}_{\mathrm{Gad}} + D^{*}_{\gamma }(\omega ), \end{aligned}$$

where $D^{*}_{\mathrm{Gad}} = 1.2 \times 10^{-10}$ m/$\hbox {s}^2$ is a fixed parenchymal gadobutrol diffusivity [16] and where $D^{*}_{\gamma }$ is a gamma-distributed random variable with shape $k=3$ and scale $\theta = 0.75 \times D^{*}_{\mathrm{Gad}}/k$. The choice of shape and scaling parameters ensures that (i) the diffusion coefficient is positive, (ii) its expected value matches reported values of parenchymal gadobutrol diffusivity [16], and (iii) its variability allows for values up to 2–3 times larger or smaller than the average with low probability. The last modelling choice reflects diffusivity values in the range 1–10 $\times 10^{-10}\hbox { m}/\hbox {s}^2$ in agreement with previous reports [26]. The probability distribution of $D^{*}$ is shown in Fig. 2.

#### Effective diffusion coefficient modelled as a random field

In order to represent spatial heterogeneity in the diffusion coefficient, we next model $D^{*}$ as a continuous random field. Again, we set

8 $$\begin{aligned} D^{*}(x,\omega ) = 0.25 \times D^{*}_{\mathrm{Gad}} + D^{*}_{f} (x, \omega ), \end{aligned}$$

where $D^{*}_{f}$ now is a random field such that for each fixed $x \in \mathcal {D}$, $D^{*}_{f}(x, \cdot )$ is a gamma-distributed random variable with the same parameters as $D^{*}$ in (7). We define this field with a correlation length of 0.01 m. By construction, spatial changes in the diffusivity occur at a length scale corresponding to the correlation length. More details are provided in Additional file 1.

### Stochastic velocity modelling

In what follows we introduce three different models for the velocity field, each representing a different hypothesis regarding intraparenchymal ISF/CSF movement. We emphasize that each model represents a homogenized velocity field averaged over physiological structures.

#### Glymphatic velocity model: arterial influx and venous efflux

To define a stochastic homogenized velocity model representing the glymphatic pathway, we assume that ISF follows separate inflow and outflow routes: entering the brain along paraarterial spaces and exiting along paravenous spaces [2]. We further suggest that

1. Substantial changes within the velocity field happen after a distance proportional to the mean distance between arterioles and venules.
2. The blood vessel structure is random and independent from the position within the parenchyma in the sense that the presence of paraarterial or paravenous spaces are equally likely at any point in space. Mathematically, this assumption requires the expected value of each of the velocity components to be zero.
3. The velocity field varies continuously in space and is divergence-free ($\nabla \cdot v = 0$), i.e. no CSF/ISF leaves the system e.g. through the bloodstream.
4. We set the expected velocity magnitude $||v|| = \sqrt{v_x^2 + v_y^2 + v_z^2}$ to be $v_{\mathrm{avg}} = 0.17$ µm/s and we allow for up to two to three times larger and up to ten times smaller values with low probability [26].

Although ISF/CSF velocities in paravascular regions may be higher [13] than what we propose, the velocity field here models an averaged bulk flow over a larger area (comprised of e.g. PVS and adjacent tissue). Bulk flow velocity magnitudes in rats have been reported to be in the range of approximately 0.1–0.24  µm/s [26, 57].

**Fig. 3:**
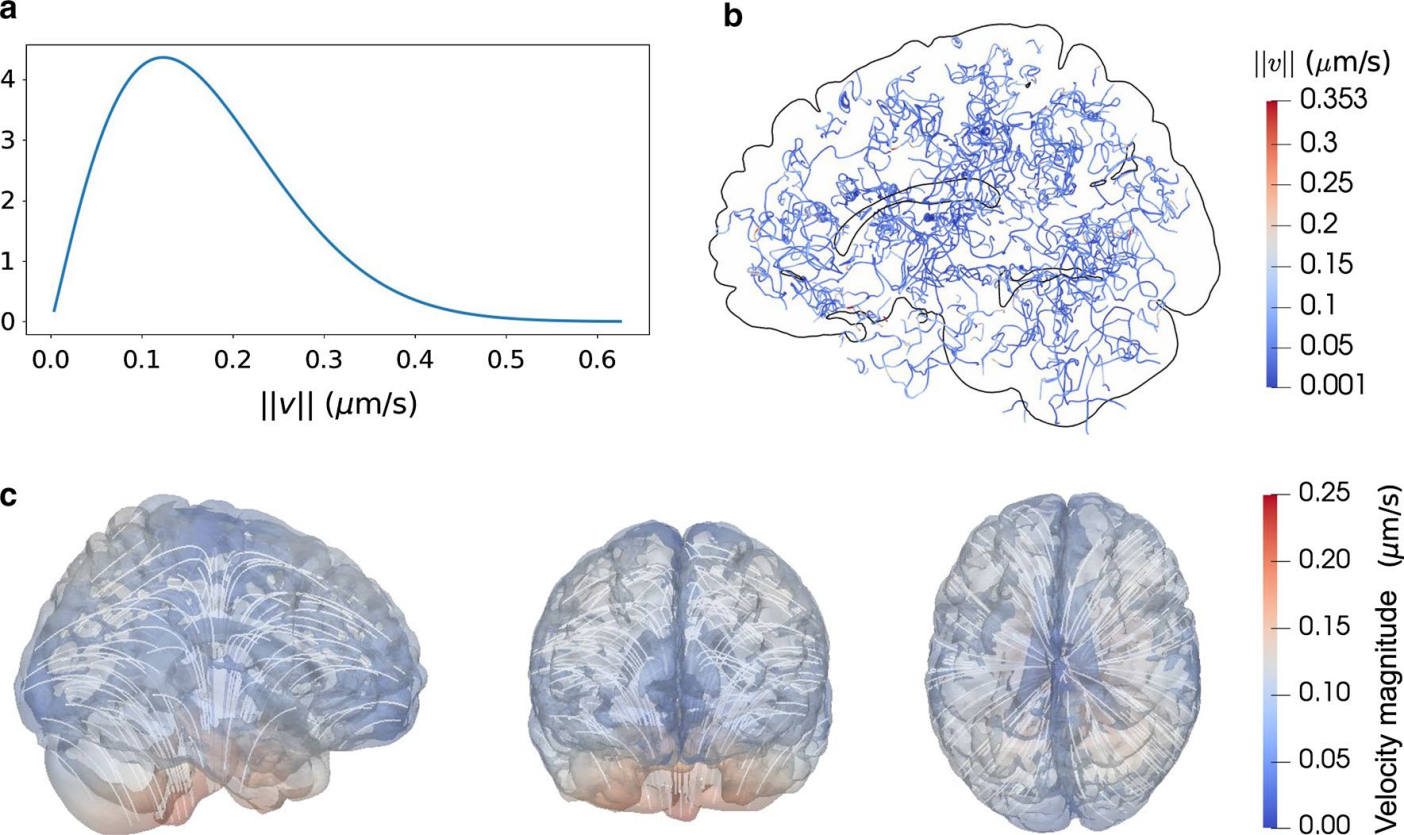
Stochastic aspects of the glymphatic circulation velocity fields (Models V1 and V2). **a** Probability density of the glymphatic circulation velocity magnitude $\Vert v \Vert$ cf. (9). **b** Streamlines of a corresponding velocity field sample. **c** Velocity magnitude and streamlines for the directional velocity field $v_{\mathrm{dir}}$ as given by (10). The flow field is assumed to follow cardiovascular pulses upwards along the brain stem. After entering the deeper parts of the brain, the bulk flow spreads out at reduced velocity magnitude. From left to right: sagittal, coronal and transverse view

To address these stipulations, we define the stochastic glymphatic circulation velocity field

9 $$\begin{aligned} v(x,\omega ) = v_{\text {avg}}\cdot \eta \ 10^{-\mathcal {E}(\omega )} \left( \nabla \times \left[ \begin{array}{c} X(x,\omega ) \\ Y(x,\omega ) \\ Z(x,\omega ) \end{array}\right] \right) , \end{aligned}$$

where $\eta$ is a scaling constant chosen such that the magnitude of *v* satisfies $\mathbb {E}[||v||^2]^{1/2} = v_{\text {avg}}$, $\mathcal {E}(\omega )$ is an exponentially distributed random variable with mean 0.2 and $X(x,\omega )$, $Y(x,\omega )$ and $Z(x,\omega )$ are standard independent identically distributed (i.i.d) Matérn fields with correlation length $\lambda = 1020$ µm. For more details, we refer the reader to Additional file 1 (Section A.3). A sample of the glymphatic circulation velocity field together with the velocity magnitude distribution is shown in Fig. 3a–b.

#### Glymphatic velocity model with additional directional velocity field

Above we assumed that the blood vessel distribution was independent of the spatial position within the parenchyma and that bulk flow from arterial to venous PVS occurs on a small length scale proportional to the mean distance between arterioles and venules. However, transport of tracer might also happen on a larger length scale along larger vascular structures present in given physical regions (such as e.g. the Circle of Willis). As CSF is hypothesized to enter the brain along penetrating arteries, the direction of cardiac pulse propagation may induce an additional large-scale directionality of the glymphatic circulation as well. The cardiac pulse follows the vessel paths of larger arteries entering the brain from below, and from there spreads out almost uniformly [58, 59]. The pulses also seem to traverse deep gray matter structures on the way up towards the ventricles.

To model such behavior, we introduce an additional large-scale directional velocity field $v_{\mathrm{dir}}$, with characteristics qualitatively similar to what is described in the literature [58, 59]:,

10 $$\begin{aligned} v_{\mathrm{dir}}(x) = -v_f \left( \begin{array}{c} \arctan (15x_1)(|x_1|-0.1) \\ \arctan (15x_2)(|x_2|-0.1) \\ -0.9x_3+0.06-\sqrt{x_1^2+x_2^2} \end{array} \right) , \end{aligned}$$

where $v_f = 2 \times 10^{-6}$ m/s. For a plot of $v_{\mathrm{dir}}$, see Fig. 3c. The velocity field $v_{\mathrm{dir}}$ induces a net flow out of the parenchyma at the very low rate of 0.007 mL/min. We superimpose this deterministic directional velocity field onto the stochastic glymphatic circulation velocity field to define the stochastic glymphatic directional velocity field:

11 $$\begin{aligned} v(x, \omega ) = v_{\mathrm{V1}}(x, \omega ) + v_{\mathrm{dir}}(x), \end{aligned}$$

where $v_{\mathrm{V1}}$ is given by (9). This velocity model thus takes into account both the “randomness” of small arteries (small-scale directionality), but also the “deterministic” presence of large arteries and possibly other structures (large-scale directionality) of blood flow propagation [58, 59] .

#### Capillary filtration model V3: arterial inflow with a homogeneous sink throughout the brain

Several independent studies demonstrate that CSF may enter the brain parenchyma along spaces surrounding penetrating arteries [2, 4, 13, 27]. However, the glymphatic efflux concept of a bulk flow of CSF through the ECS and recirculation into the SAS through paravenous spaces has been severely questioned [4, 7, 31, 60]. As a variation, we here therefore also consider a stochastic velocity model representing paraarterial influx without a direct return route to the CSF. Instead, we assume that ISF/CSF is drained inside the brain parenchyma along some alternative efflux pathway. This pathway may include the capillaries or separate spaces along the PVS directly into cervical lymph nodes.

In light of this, we consider the following alternative velocity assumptions. (1) There is a net flow of CSF into the brain and (2) ISF is cleared within the parenchyma via some, here unspecified, route. For instance, it has been proposed that production and absorption is present all over the CSF system and that capillaries and ISF continuously exchanges water molecules [61]. However, drainage of large molecules through this route is unlikely as capillary endothelial cells are connected by tight junctions [7]. It has also been reported that lymph vessels may be capable of also draining larger molecules from brain tissue into deep cervical lymph nodes, possibly through paravenous spaces [62]. In addition, other outflow routes may exist, including clearance by degradation or meningeal lymphatic vessels [63].

**Fig. 4:**
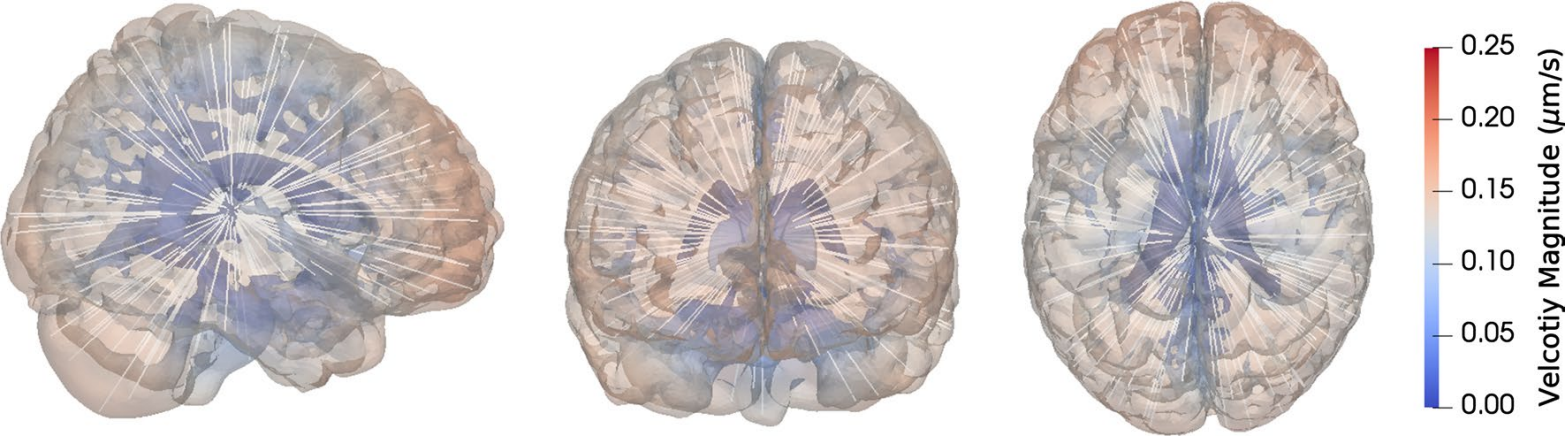
Sample Model V3 velocity field. Velocity magnitude and streamlines for the velocity field as given by (12). Flow is assumed to occur from the cortex towards the ventricles with reduced velocity magnitude along the way due to clearance. From left to right: sagittal, coronal and transverse view

To address these assumptions, we define a stochastic arterial inflow velocity field as a radially symmetric field pointing inwards from the SAS interface to the brain region around the lateral ventricle. This central region is modelled in what follows as a sphere of radius $R = 8$ cm and center given by $x_c$ in the lateral ventricles. Mathematical experimentation lead to the following *ansatz* for such velocity:

12 $$\begin{aligned} v(x, \omega ) =\bar{v}(\omega )\exp \left( -\frac{3(R-||x-x_c||)^2}{R^2 -(R-||x-x_c||)^2}\right) (x_c-x), \end{aligned}$$

where $\bar{v}(\omega )$ is a gamma random variable chosen such that the probability distribution of the velocity magnitude is comparable to that of the glymphatic circulation velocity defined by (9). The shape parameter $k=2$ and the scale parameter is set such that again $\mathbb {E}[||v||^2]^{1/2} = v_{\mathrm{avg}}$. Note that in this case, the expected value of the velocity components are non-zero. To satisfy (2), we model the drainage of tracer by setting $r = 1 \times 10^{-5}\,\hbox {s}^{-1}$, which typically results in $40 \%$ drainage of the injected tracer over 48 h. An example of the velocity field given by (12) is shown in Fig. 4.

### Quantities of interest, random sampling and uncertainty analysis

#### Quantities of interest

To evaluate the speed and characteristics of tracer movement into and in the brain parenchyma, we consider a set of output quantities of interest. Each quantity of interest $Q = Q(\omega )$ depends on the event $\omega$ via $c(\cdot , \cdot , \omega )$ as defined by (1).

To quantify the overall spread of tracer in the gray and white matter, we consider the (integrated) amount of tracer in the gray matter $Q_g$ and in the white matter $Q_w$ at time points $\tau$:

13 $$\begin{aligned} Q_g(\omega ) = \int _{D_g} c(\tau , x, \omega ) \, \mathrm {d} x, \quad Q_w(\omega ) = \int _{D_w} c(\tau , x, \omega ) \, \mathrm {d} x. \end{aligned}$$

We pay particular attention to the times $\tau \in \{3, 5, 8, 24\}$ h. To further differentiate, we also defined two localized quantities of interest at each time $\tau$: the average tracer concentration $q_g$ in a small subregion of the gray matter $S_g$ and analogously $q_w$ for a small subregion of the white matter $q_w$:

14 $$\begin{aligned} q_{g} = \frac{1}{V_g} \int _{S_g} c(\tau , x,\omega ) \, \mathrm {d} x, \quad q_{w} = \frac{1}{V_w} \int _{S_w} c(\tau , x,\omega ) \, \mathrm {d} x, \end{aligned}$$

where $V_g$ and $V_w$ is the volume of the gray and white matter subregions, respectively. The size and relative location of the subregions $S_g$ and $S_w$ within the computational domain are illustrated in Fig. 1. To further quantify the speed of propagation, we define the white matter activation time $F_w$:

15 $$\begin{aligned} F_{w}(\omega ) = \left\{ \min t \, | \, \int _{\Omega _w} c(t, x,\omega ) \, \mathrm {d} x/ n_0 > X \right\} , \end{aligned}$$

where $n_0$ is the total amount of tracer injected into the SAS (0.5 mmol) and *X* is a given percentage. Given the time course of the expected tracer distribution to the white matter [16], we here chose $X = 10 \%$. Finally, we also define the analogous regional (white matter) activation time

16 $$\begin{aligned} f_w(\omega ) = \left\{ \min t \, | \, \frac{1}{V_w}\int _{S_w} c(t, x,\omega ) \, \mathrm {d} x> Y \right\} , \end{aligned}$$

where $$Y = 10^{-3}$$ mol/$$\hbox {m}^3$$

For plotting the boundary tracer concentration over time, we define three axial planes along the z-axis ($z = -0.1, 0, 0.1$ m) to represent the level of the foramen magnum, Sylvian fissure and precentral sulcus, respectively.

#### Random sampling and uncertainty analysis

We consider the six output quantities of interest: the amounts of tracer in gray and white matter at given times (13), the average tracer concentrations in subregions of gray and white matter (14), the white matter activation time (15), and the white regional activation time (16) for all 5 stochastic model variations.

To sample a quantity of interest from its distribution, we first compute a sample of each of the random coefficients in (1) from their distribution, second, solve (1) for *c* with the given coefficient sample, and third, evaluate the quantity of interest with the computed solution. The random diffusion and velocity coefficient fields were sampled using the sampling technique as described in e.g [54]. We used the standard Monte Carlo approximation cf. (6) to compute an estimate $\hat{Q}$ of each expected quantity of interest value $\mathbb {E}[Q]$ using $N = 3200$ samples. The statistical error introduced by this approximation decreases with $O(N^{-1/2})$. The choice $N = 3200$ ensures that $3 (\hat{V}/N)^{1/2} < 0.01 \hat{Q}$, where $\hat{V}$ is the sample variance of $\hat{Q}$. For each output quantity of interest, we also estimate its probability distribution, from which we compute $99.73\%$ prediction intervals for each $\hat{Q}$. A prediction interval is a statistical term that roughly indicates that if we were to take a new sample (i.e. a new simulation) of *Q*, there would be a $99.73\%$ chance for this sample to fall within the interval.

### Numerical methods and implementation

The diffusion–convection Eq. (1) was solved numerically using a finite element method with continuous piecewise linear finite elements in space, and an implicit midpoint finite difference discretization time with time step $\Delta t = 15$ min, combined with mass lumping [64]. The finite element mesh $\mathcal {T}_h$ was an adaptively refined version of the gray and white matter of the Colin27 human adult brain atlas mesh [42] version 2 with 1,875,249 vertices and 9,742,384 cells. An outer box of dimensions $0.16 \times 0.21 \times 0.17$ ($\hbox {m}^3$) with mesh size 0.0023 m was used for the sampling of the Gaussian fields.

For the models with non-zero velocity (Models V1, V2, V3), (1) was typically mildly convection-dominated with an upper estimate of the Péclet number of

17 $$\begin{aligned} Pe \approx \frac{9 L v_{\mathrm{avg}}}{D^{*}_{\mathrm{Gad}}} \approx O(10^3), \end{aligned}$$

where $L \approx 0.084$ m is half the diameter of the computational domain, $v_{\mathrm{avg}} = 0.17$ µm/s, and $D^{*}_{\mathrm{Gad}} = 1.2 \times 10^{-10}$ m/$\hbox {s}^2$. The boundary condition (5) was discretized explicitly in time using the trapezoidal rule, making the overall scheme first-order in time and second order-in space. For more details, we refer to Additional file 1 (Section B).

The numerical solver was implemented in Python using the FEniCS finite element software [65] and previously verified in-house parallel Monte Carlo routines [54]. The extended box mesh was created using the Gmsh software [66]. The linear system was solved using the PETSc [67] implementation of the GMRES algorithm preconditioned with the BoomerAMG algebraic multigrid algorithm from Hypre [68]. The numerical solver was verified using a convergence test comparing different mesh refinements, time steps, and stabilization techniques, including SUPG [69], for a set of deterministic numerically worst-case models (with large velocities and small diffusion coefficients) [see Additional file 1 (Section C)]. We used Matplotlib (version 2.1.1) and Paraview (version 5.4.1) for visualization.

## Results

### Non-random diffusion as a baseline for parenchymal solute transport

**Fig. 5:**
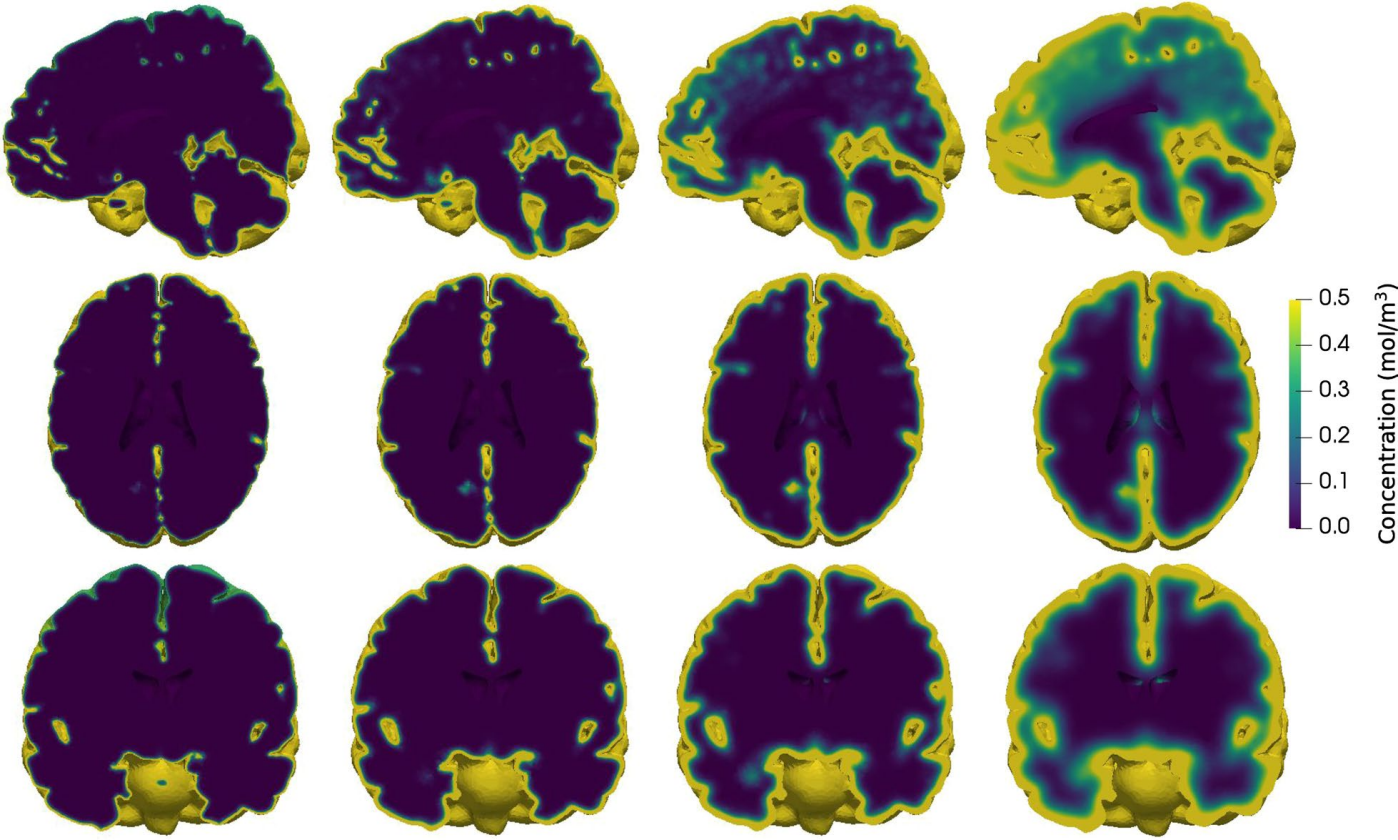
Baseline tracer evolution. Parenchymal tracer concentration after (from left to right) 1, 3, 8 and 24 h of diffusion in (from top to bottom) sagittal, transverse and coronal planes. Initially, most of the tracer is found in inferior regions. At 24 h, tracer has penetrated substantially into the gray matter, but not into the deep, central regions

To establish a baseline for parenchymal solute transport, we first simulated the evolution of a tracer spreading in the SAS and in the parenchyma via diffusion only, using a constant (i.e. non-random) effective diffusion coefficient for gadobutrol ($D^*= 1.2 \times 10^{-10}\,\hbox {m}^2$/s). The resulting parenchymal tracer spread over 24 h is shown in Fig. 5. The tracer concentration increases first in inferior regions and in the gray matter. Tracer does not penetrate deep into white matter regions within this time frame. Slower penetration into white matter is expected as the white matter is located further from the outer brain surface. In the sagittal plane (top), tracer enhancement is more prominent than in the other two plane as the sagittal plane shown is close to the CSF-filled longitudinal fissure.

**Fig. 6:**
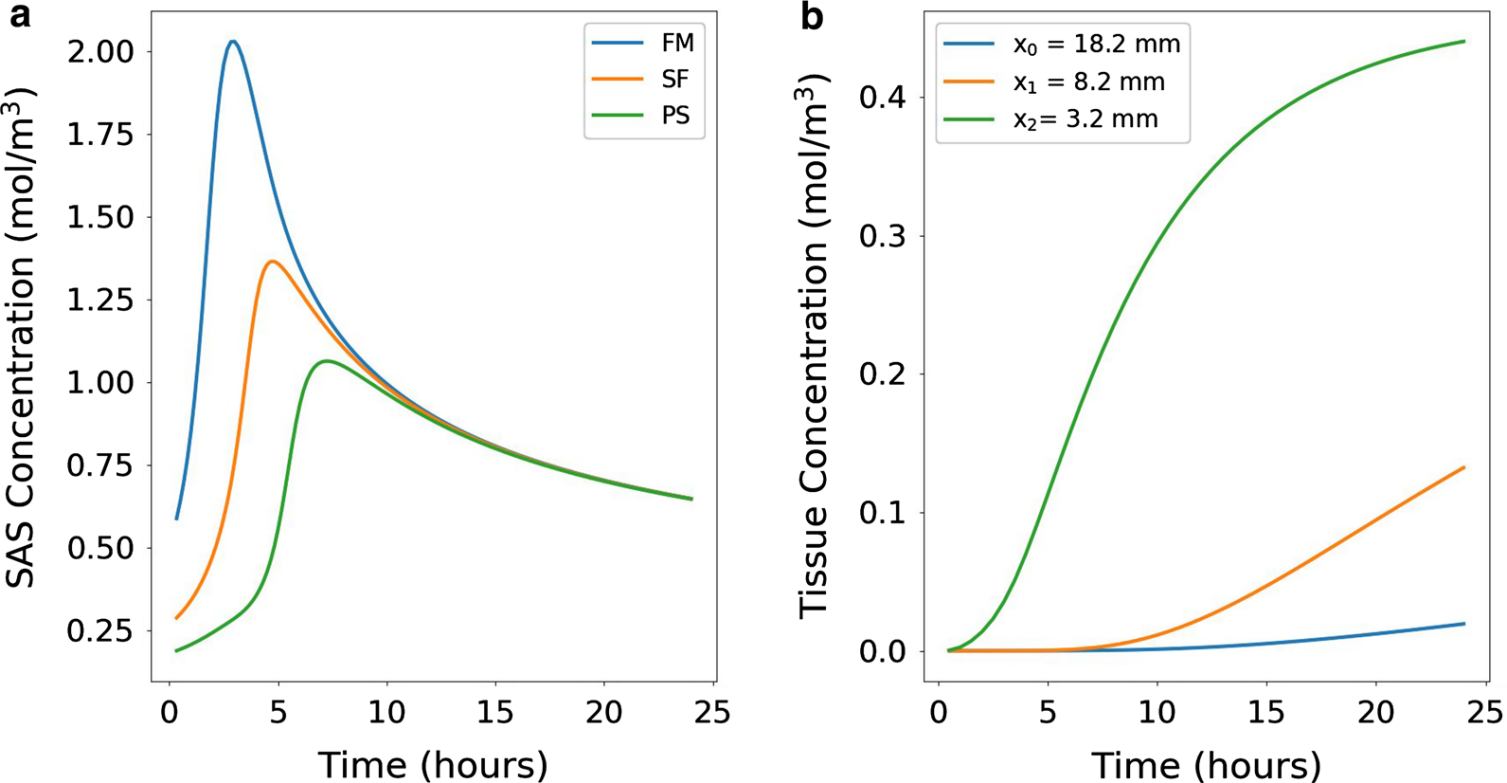
Tracer concentrations. **a** Tracer concentration in the SAS ($c_{\mathrm{CSF}}$) used as boundary conditions at the brain surface at the level of the foramen magnum (FM), Sylvian fissure (SF) and the precentral sulcus (PS). At the lower level of the SAS, tracer concentration peaks at around 3 h, while at the upper levels, peak concentration occurs later. Following peak values, the concentration in the SAS decreases as tracer enters the parenchyma. The SAS concentration is modeled by (4).** b** Tracer concentration over time in three different points at a given distance from the brain surface. The points were chosen along a line directly from the cortex towards the ventricles at the level of the Sylvian fissure

Figure 6a shows the boundary tracer concentration (concentration in the SAS) over time at the levels of the foramen magnum ($z=-0.1$ m), Sylvian fissure ($z=0$ m) and precentral sulcus ($z=0.1$ m). During the first few hours, boundary tracer concentration at the level of the foramen magnum increases rapidly, and peaks at 3 h reaching approximately 2.0 mol/$\hbox {m}^3$. Boundary tracer concentrations close to the Sylvian fissure and precentral sulcus are lower, and the time to reach peak concentrations is longer. For the Sylvian fissure, peak concentration in the CSF is 1.4 mol/$\hbox {m}^3$, at 5 h, while the precentral sulcus concentration reaches 1.1 mol/$\hbox {m}^3$ at 7 h. We note that as the boundary condition depends on the parenchymal tracer concentration itself [cf. (5)], the boundary tracer concentration will differ slightly in subsequent simulation setups.

In Fig. 6b, concentration profiles are shown for three interior points at different distances from the brain surface. The points were chosen along a line from the brain surface towards the ventricles at the height of the Sylvian fissure (z = 0). The tracer concentration at these points stays low for the first few hours before steadily increasing. For the point closest to the SAS ($x_2$), the concentration rises faster than for the other two points, and is almost equal to the SAS concentration at 24 h (0.4 vs 0.5 mol/$\hbox {m}^3$). In the middle point ($x_1$), tracer concentration starts increasing after 6–7 h and reaches approximately 0.15 mol/$\hbox {m}^3$ after 24 h. For the most interior point ($x_0$), tracer concentration starts and stays low throughout the 24 h time span. At 24 h, the tracer concentration in all three points is still increasing.

### Quantifying the effect of uncertainty in effective diffusion magnitude

We first aimed to quantify the effect of uncertainty in the magnitude of the effective diffusion coefficient on the time evolution of tracer in the gray and white matter. In particular, we computed the tracer concentration, together with auxiliary output quantities, evolving via diffusion only with a gamma-distributed random variable diffusion coefficient (Model D1).

**Fig. 7:**
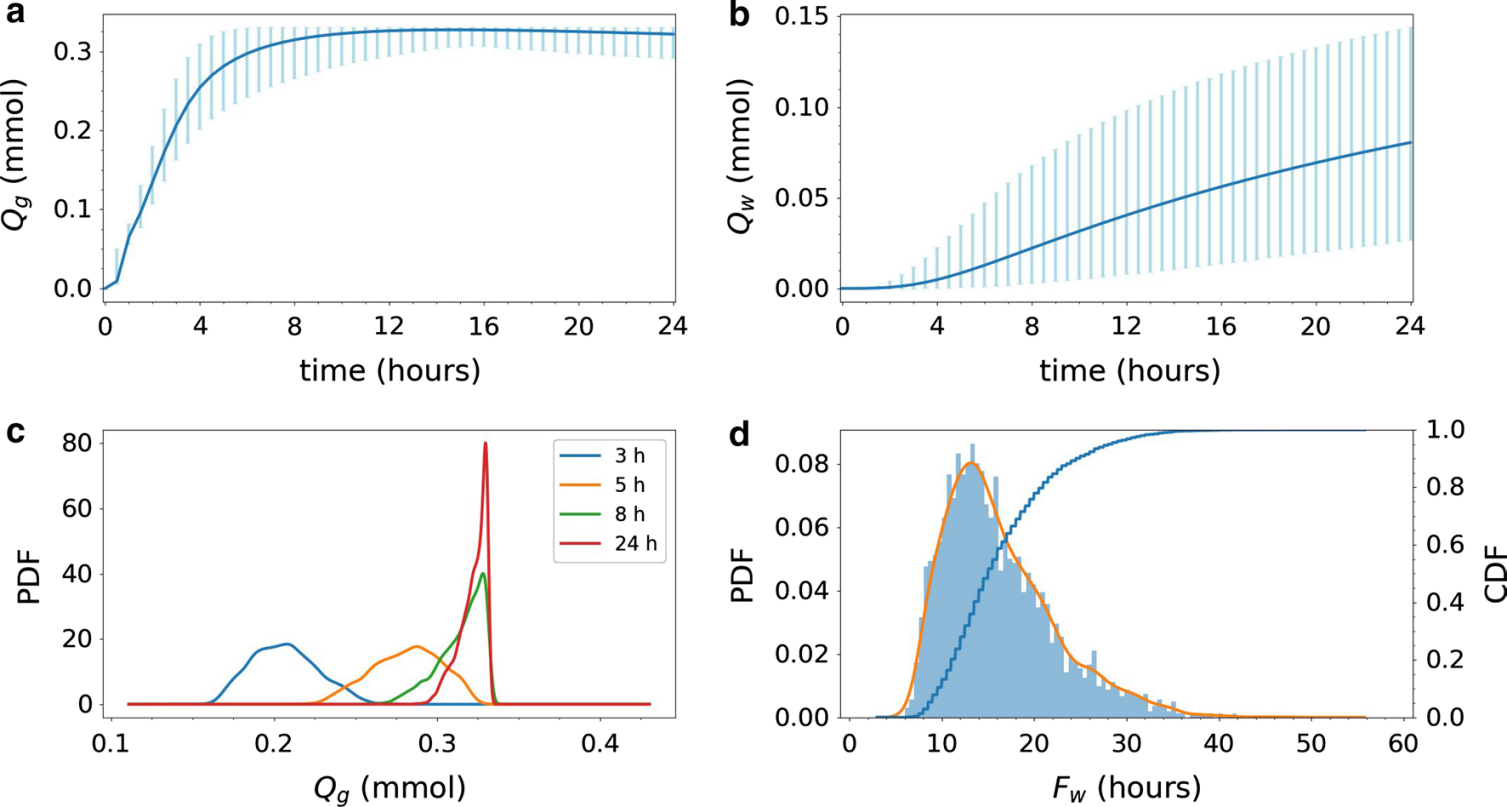
Uncertainty quantification for Model D1. The integrated amount of tracer in the** a** gray matter $Q_g$ and** b** white matter $Q_w$ over time; $Q_g$ and $Q_w$ as defined by (13). The blue curves show the expected value. The light blue vertical bars indicate the variability: 99.73% of the samples fall within the plotted range (with 0.135% of the samples above and 0.135% below).** c** The probability density functions (PDFs) corresponding to $Q_g$ at 3, 5, 8 and 24 h after tracer injection.** d** Histogram of white matter activation time $F_w$ as defined by (15) (bars), corresponding estimated PDF (orange curve), and corresponding cumulative density function (CDF). Uncertainty in the magnitude of the effective diffusion coefficients substantially impact the amount of tracer found in the gray and white matter and the white matter activation time

The amount of tracer found in the gray and white matter differ both in magnitude and variation (Fig. 7a–c). The expected amount of tracer in the gray matter increases rapidly, and doubles from 1 to 2 h (0.065 to 0.13 mmol), and again from 2 to 4 h (0.13 mmol to 0.25 mmol). The gray matter reaches a peak after approximately 15 h, while the white matter did not reach steady steady within 24 h. There is substantial variation in the amount of tracer in gray matter throughout the 24 h time span. The variation is at its largest between 2 and 8 h where the length of the 99.73%-intervals range from 0.064 mmol to 0.11 mmol corresponding to 13–22% of the total tracer injection of 0.5 mmol. Ultimately, the amount of tracer will reach a steady-state solution, constant in space and time, independently of the diffusion coefficient. Therefore, after a certain point in time, variation decreases as all solutions converge towards the same steady state. The changes in variation of tracer found in the gray matter over the 24 h are also illustrated by the change in the estimated probability density function (PDF) of the total amount of tracer at a given time (Fig. 7c). After 3 and 5 h (blue and orange curve) the PDFs are symmetric, and with more spread for the later time point. As time evolves, the PDFs become more left skewed (green and red curve), as in almost all cases, the concentration approaches but never surpasses the steady state value.

The amount of tracer in the white matter changes slowly for the first 2 h, before starting to increase after 3–4 h (Fig. 7b). After 4 h, the expected amount of tracer in the white matter is only 0.0048 mmol, increasing to 0.022 mmol after 8 h, and 0.056 mmol after 16 h. The variation is substantial and increasing with time: the length of the 99.73%-interval is 0.022 mmol at 4 h, 0.065 mmol at 8 h and 0.10 at 16 h. At 24 h, the uncertainty in diffusion coefficient may explain a factor of approximately 5 in deviation from the lowest (0.027 mmol) to the highest (0.14 mmol) predicted amount of tracer in the white matter.

The estimated PDF and cumulative density function (CDF) for the white matter activation time (i.e. time for 10% of tracer to reach the white matter) is shown in Fig. 7d. We observe that the most likely white matter activation time is approximately 14 h. The white matter activation time is less (than 10%) likely to be less than 9.5 h, but (more than 90%) likely to be less than 24.5 h. The activation time may exceed 24 h, but is highly unlikely to go beyond 40 h (CDF > 0.998). The white matter activation threshold was reached in all samples within the simulation time span.

### Quantifying the effect of uncertainty in diffusion heterogeneity

Brain tissue is heterogeneous [55], varies from individual to individual, and is clearly not accurately represented by a single diffusion constant. To further investigate the effect of uncertainty in the diffusion coefficient and in particular to study the effect of spatial heterogeneity, we modelled the diffusion coefficient as a spatially-varying random field (Model D2).

The amounts of tracer found in gray and white matter for Model D2 are nearly identical to those resulting from Model D1 in terms of expected value (data shown later cf. Fig. 10), but with substantially less variability. The length of the 99.73% prediction interval for amount of tracer in gray matter ($Q_g$) is less than 0.0071 mmol for all times after the first half hour, corresponding to a relative variability (compared to the expected value) of between 2.2 and 10.9% throughout the 24 h time span. For white matter, the length of the 99.73% prediction interval is increasing with time, with the relative variability at 24 h at 7.9%.

**Fig. 8:**
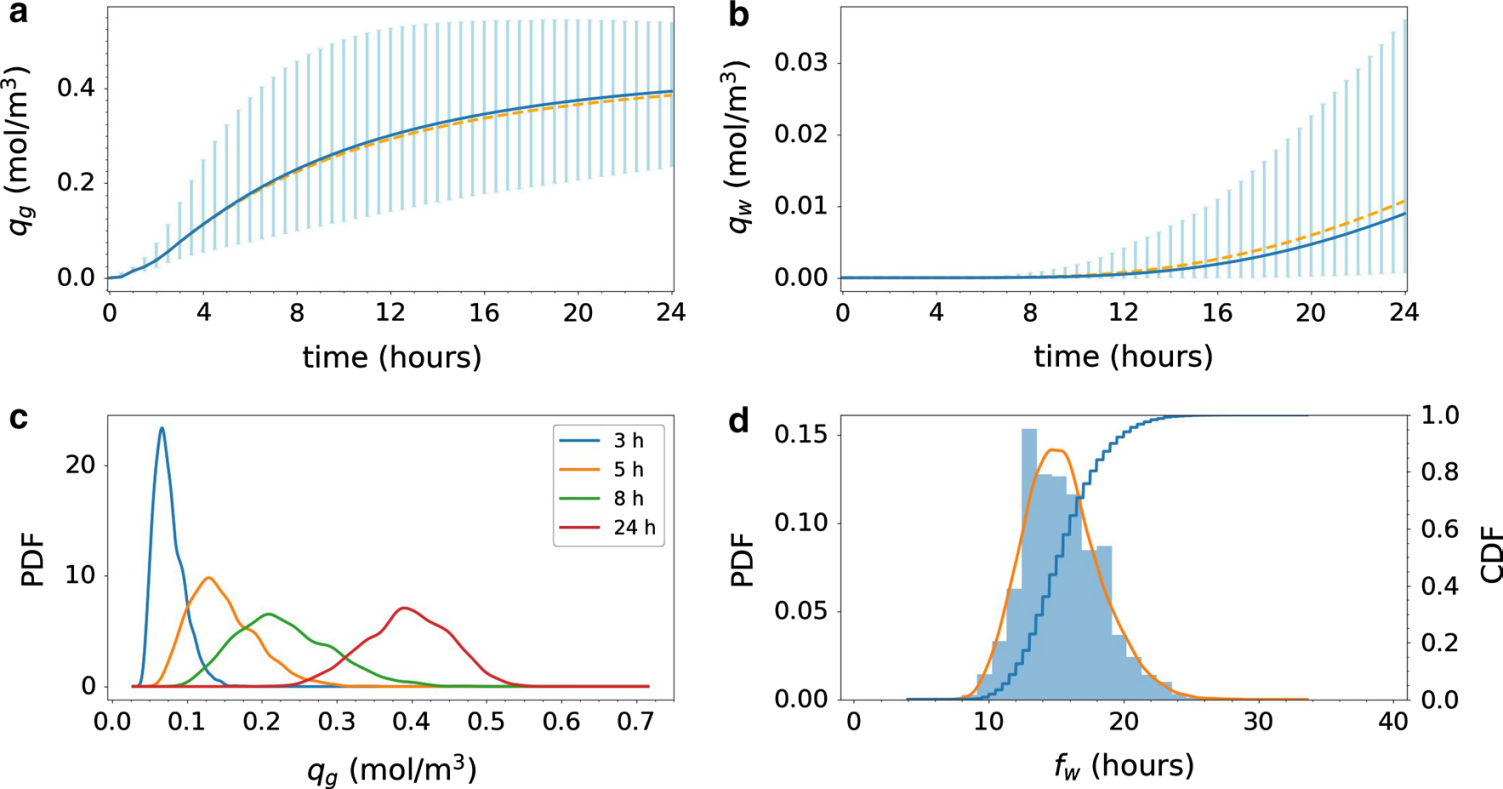
Uncertainty quantification for Model D2. The average tracer concentration in a subregion of **a** gray matter $q_g$ and **b** white matter $q_w$ as defined by (14). The blue curves show the expected value. The light blue vertical bars indicate the variability: 99.73% of the samples fall within the plotted range (with 0.135% of the samples above and 0.135% below). The dashed orange lines in **a** and **b** indicate the analogous expected value curve resulting from Model D1 (constant diffusion only), for comparison. **c** The probability density functions (PDFs) corresponding to $q_g$ at 3, 5, 8 and 24 h after tracer injection. **d** Histogram of white subregion activation time $f_w$ as defined by (16) (bars), corresponding estimated PDF (orange curve), and corresponding cumulative density function (CDF). Uncertainty in the heterogeneity of the diffusion coefficient leads to a wide range of likely average tracer concentrations in the white matter throughout the time span

When considering the average concentration of tracer in two smaller regions of interest [cf. (14)], variability in model D2 increases drastically (Fig. 8). In the gray matter region (Fig. 8a), the expected average tracer concentration increases steadily to 0.11 mol/$\hbox {m}^3$ after 4 h, 0.23 mol/$\hbox {m}^3$ after 8 h, 0.35 mol/$\hbox {m}^3$ after 16 h and is still increasing after 24 h. The variability is moderate after 3 h (Fig. 8c), but increases thereafter. The length of the 99.73% prediction interval peaks at 0.39 mol/$\hbox {m}^3$ after 11 h before decreasing moderately for later times.

The expected average tracer concentration in the white matter is low, lower than in the gray matter (Fig. 8b) by a factor of at least 40, and starts increasing only after approximately 14 h. For the samples in the lower range of the 99.73% interval (thus with the lower effective diffusivity), the concentration in the white matter region remains close to zero after 24 h. For the white region activation time, we observe some variability (Fig. 8d): the peak likelihood is after 14–15 h, less (than 10%) likely to be less than 12 h, and (more than 90%) likely to be less than 19 h. The white subregion activation threshold was reached in all samples within the simulation time span.

### Quantifying the effect of glymphatic circulation

In light of the substantial uncertainty surrounding ISF/CSF flow in paravascular/perivascular spaces and potential ISF flow in extracellular spaces, we now turn to study the effect of uncertain velocity fields. To investigate the effect of uncertainty in a glymphatic velocity model, we defined a random velocity field with correlation length corresponding to the typical distance between parenchymal arterioles and venules (Model V1).

The expected amounts of tracer found in the whole gray and whole white matter for Model V1 are nearly identical to those found for Model D2 and Model D1, while the variability is minimal (data shown later cf. Fig. 10). In response, additional Monte Carlo simulations using up to three times higher values of the velocity magnitude average were performed, which did not change the expected value (data not shown). The only difference was a slight increase in variability. Thus, on average, small random variations in fluid velocity did not increase (or decrease) the tracer distribution into the parenchyma on a global scale. This observation can be interpreted in the light of the small correlation length of the velocity field compared to the size of the whole gray and white matter.

**Fig. 9:**
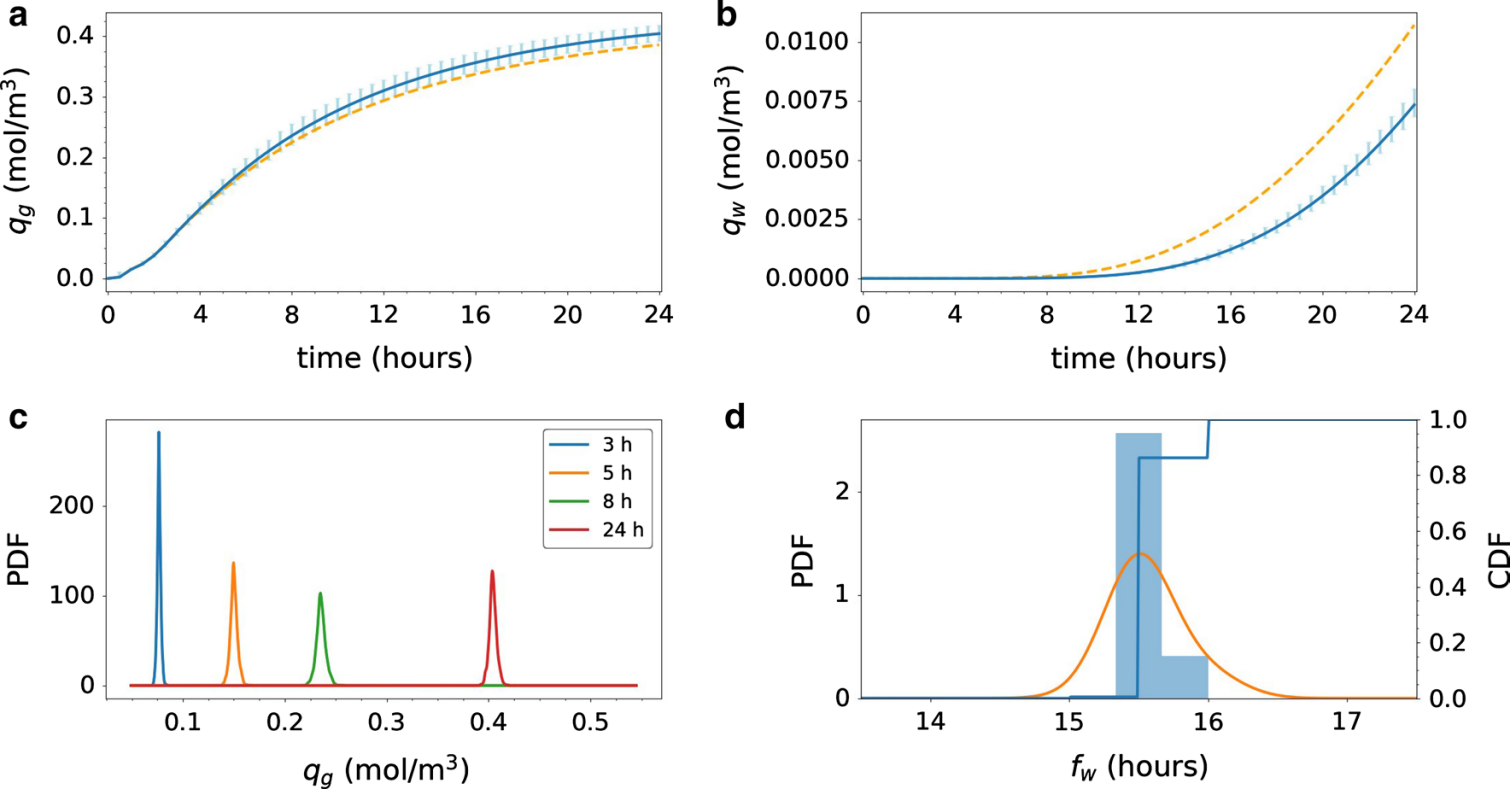
Uncertainty quantification for Model V1. The average tracer concentration in a subregion of **a** gray matter $q_g$ and **b** white matter $q_w$ as defined by (14). The blue curves show the expected value. The light blue vertical bars indicate the variability: 99.73% of the samples fall within the plotted range (with 0.135% of the samples above and 0.135% below). The dashed orange lines in **a** and **b** indicate the analogous expected value curve resulting from Model D1 (constant diffusion only), for comparison. Expected values for $q_g$ are nearly identical as for Model D1 and D2, but variation is much lower. Expected values for $q_w$ are lower than for Model D1 and variation is much lower (**c**). The probability density functions (PDFs) corresponding to $q_g$ at 3, 5, 8 and 24 h after tracer injection. The PDFs show very low variation. Variation increases slightly over time. **d** Histogram of white subregion activation time $f_w$ as defined by (16) (bars), corresponding estimated PDF (orange curve), and corresponding cumulative density function (CDF)

The expected average tracer concentration in the gray subregion $q_g$ reaches 0.2 mol/$\hbox {m}^3$ in 7 h (Fig. 9a). This is a considerable amount of time, given that the initial average SAS concentration is 3.57 mol/$\hbox {m}^3$. The expected average tracer concentration in the white subregion $q_w$ is lower, and only reaches 7.3 mmol/$\hbox {m}^3$ in 24 h (Fig. 9b). We observe that the expected $q_g$ increases marginally faster with the glymphatic velocity model than for pure diffusion: at 24 h, $q_g$ is 2.5% higher for V1 (0.40 mol/$\hbox {m}^3$) than for D1 (0.39 mol/$\hbox {m}^3$). On the other hand, the expected $q_w$ increases faster with pure diffusion than with the glymphatic velocity model: at 24 h, $q_w$ is 34% lower for V1 (0.0073 mol/$\hbox {m}^3$) than for D1 (0.011 mol/$\hbox {m}^3$). The peak relative difference between pure diffusion and the upper limit of the 99.73% interval of model V1 is high after 1 h, due to low tracer concentration overall. The next peak occurs after 8 h where the relative difference is 13% between the two.

However, the variation in both gray and white local average tracer concentration is small. For early time points (up to 3–4 h), nearly no variation is evident in the average tracer concentration of the local regions (Fig. 9a–c). The peak length of the 99.73% interval for $q_g$ is 0.035 mol/$\hbox {m}^3$ (at 9 h), and the relative variability ranges from 6–19% in the 24 h time span. Moreover, the activation time $f_w$ shows low variability: all simulations resulted in an activation time of 15.5–16 h (Fig. 9d). The substantially reduced variability for V1 compared to e.g. D2 combined with the comparable expected values yields much larger likely sample ranges for D2 than for V1.

### Quantifying the effect of glymphatic directionality

The cardiovascular pulse propagates along the larger arteries entering the brain from below before spreading outwards [58, 59]. To assess whether and how such a directionality in the glymphatic system affects parenchymal tracer distribution, we added a net flow field to the random velocity field representing the glymphatic circulation (Model V2).

**Fig. 10:**
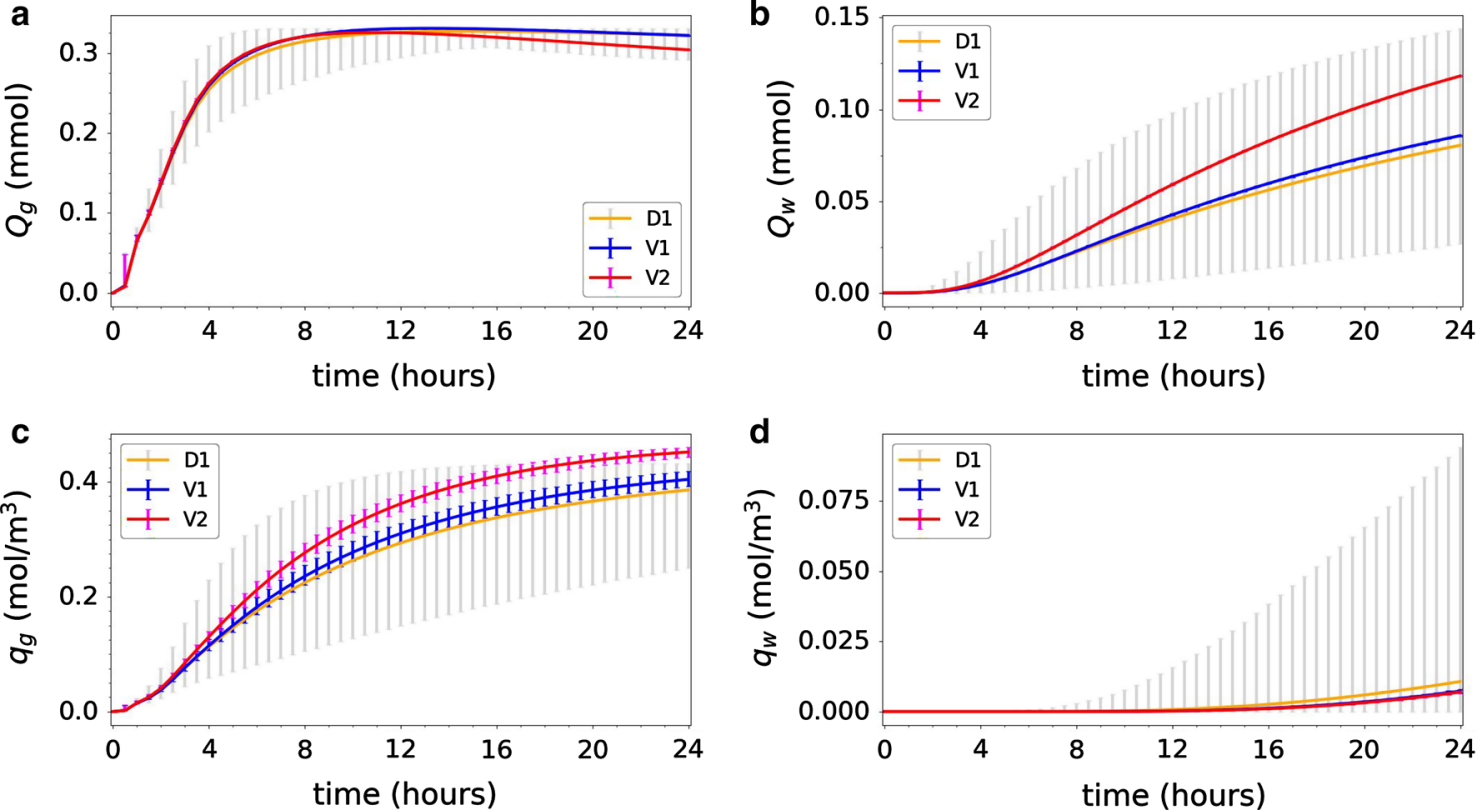
Uncertainty quantification for Model V2. Model V2 (red) in comparison with Models D1 (orange) and V1 (blue). The integrated amount of tracer in the **a** gray matter $Q_g$ and **b** white matter $Q_w$, as defined by (13), over time. The average tracer concentration in a subregion of **c** gray matter $q_g$ and **d** white matter $q_w$, as defined by (14), over time. The curves show the expected values while vertical bars indicate the 99.73% prediction intervals of the different models

With more fluid entering the brain from below, as illustrated by the streamlines of Fig. 3c, the total parenchymal amount of tracer increases. For the expected amount of tracer in gray matter, however, Model V2 was in very good agreement with Models D1 and V1 (Fig. 10a). After 13 h, the amount of tracer found in the gray matter is higher for Model D1 than for Model V2. In Model V2, more of the tracer is found deeper in the gray matter and eventually moves to the white matter. We note that the uncertainty associated with the velocity fields barely affects the amount of tracer in the gray and white matter, as demonstrated by the nearly vanishing variation associated with $Q_g$ and $Q_w$ for Model V2 (and V1) (Fig. 10a, b).

The expected amount of tracer in the white matter $Q_w$ increases substantially by the introduction of the directional velocity field (Fig. 10b). The expected value curve starts deviating from the other models after 4–5 h, and the difference increases with time. At 24 h, the expected amount of tracer found in the white matter $Q_w$ is 50% larger for Model V1 (0.12 mmol) as for Model D1 (0.08 mmol). However, in view of the large variability associated with $Q_w$ for Model D1 and the nearly vanishing variability associated with Model V2, the expected amount of white matter tracer for Model V2 falls well within the 99.73% prediction interval for Model D1.

The directional velocity field also induces an increase in the expected average tracer concentration in the gray subregion $q_g$ (0.45 mol/$\hbox {m}^3$ vs 0.40 for V1 and 0.39 mmol/$\hbox {m}^3$ for D1 at 24 h, Fig. 10c). In contrast to for $Q_g$ and $Q_w$, this quantity of interest also displays some variability, with a peak variability (0.031 mol/$\hbox {m}^3$ i.e. 10%) at 8–10 h after injection. Notably, after 21–22 h, the average tracer concentration in gray matter is larger than for pure diffusion (and for no net flow) also in terms of 99.73% prediction intervals. For $q_w$, Model V1 and V2 are in close agreement, both with distinctly less variability than Model D1 (Fig. 10d).

### Quantifying the effect of paraarterial influx with drainage

A number of open questions remain in the context of glymphatic and paravascular efflux routes. To further investigate potential pathways, we also considered a model representing paraarterial influx combined with parenchymal ISF drainage (Model V3).

**Fig. 11:**
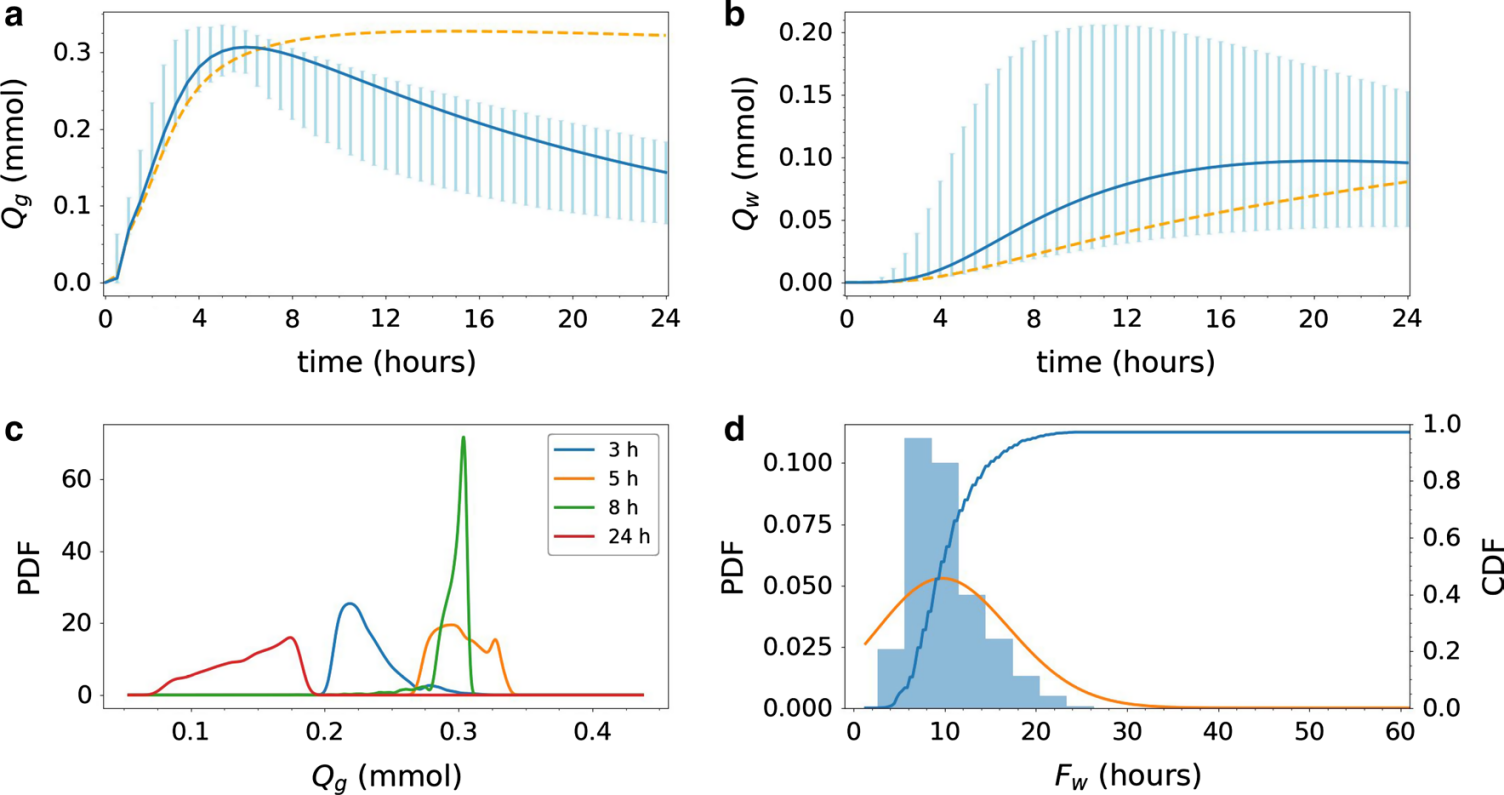
Uncertainty quantification for Model V3. The integrated amount of tracer in the **a** gray matter $Q_g$ and **b** white matter $Q_w$ over time; $Q_g$ and $Q_w$ as defined by (13). The blue curves show the expected value. The light blue vertical bars indicate the variability: 99.73% of the samples fall within the plotted range (with 0.135% of the samples above and 0.135% below). The dashed orange lines in **a** and **b** indicate the analogous expected value curve resulting from Model D1 (constant diffusion only), for comparison. Large variations in the white matter is found depending on the inflow velocity. **c** The probability density functions (PDFs) corresponding to $Q_g$ at 3, 5, 8 and 24 h after tracer injection. **d** Histogram of white matter activation time $F_w$ as defined by (15) (bars), corresponding estimated PDF (orange curve), and corresponding cumulative density function (CDF). We note that the CDF peaks at 0.96 (< 1.0) as some samples never reached the white region activation threshold

Paraarterial inflow with drainage increases the amount of tracer found in the parenchyma for the early time points (Fig. 11). After 4 h, with the lowest velocities, the amount of tracer in the gray matter is equal to models with only diffusion (0.25 mmol). With higher velocities, however, the amount of tracer found in the gray matter increases by 32% to reach 0.33 mmol. After a peak at 6–8 h, drainage and transport into white matter cause a decrease in the expected amount of tracer in the gray matter, while its variation stays more or less constant (0.11–0.12 mmol). The PDFs of the amount of tracer found in the gray matter thus have different characteristics than the two previous models, in particular the red curve (24 h) shows lower amounts of tracer than at the two previous time points.

For the white matter, the expected amount of tracer increases with time, rapidly in comparison with pure diffusion, and seems to peak at approximately 0.097 mmol (at 19–22 h) before slowly decreasing. Variation, on the other hand, is substantial and in some cases the amount of tracer found in the white matter reaches 0.2 mmol, which higher than what is seen in any previous model. This is visible by a peak of the maximum values within the 99.73% interval after 11–12 h. In Model V3, tracer is drained out of the system and the amount of tracer in the white matter is similar as for the previous models at 24 h.

The white matter activation time is likely lower for Model V3 compared to previous models, and the variation is substantial (Fig. 11d). The white matter activation time is less (than 10%) likely to be less than 6 h, but (more than 90%) likely to be less than 16.5 h. Note that the white matter activation threshold was not reached in 3% of the samples.

## Discussion

In this study, we have investigated the variability in parenchymal tracer enhancement resulting from uncertainty in diffusion and convection parameters. We designed five computational models representing different diffusion and convection regimes and used stochastic analysis to rigorously evaluate the resulting probability distributions.

In all models, 10% of the tracer reached the white matter within 40 h, with more variability in activation time for diffusion models and less variability for models including a convective velocity. Indeed, uncertainty in the diffusion parameters had a substantial impact on the amount of tracer in gray and white matter, and on the average tracer concentration in gray and white subregions. Overall, diffusion was not sufficient, with high likelihood, to transport tracer deep into the parenchyma.

A stochastic velocity field representing the glymphatic theory (with small-scale directionality only) did not increase transport into any of the regions considered, unless augmented with an additional net flow with a prescribed large-scale directionality. In the latter case, transport was increased with overwhelming likelihood: for model V2, the entire 99.73% prediction interval for the gray subregion average tracer concentration was higher than for model D1. Models including parenchymal drainage displayed substantial variability, and reached peak values for the expected amount of tracer both in gray and white matter within 24 h.

### Comparison with previous work

Our models mimic the experimental set-up of an MRI study of parenchymal tracer distribution after intrathecal gadobutrol injection [15]. In our simulations, as in the MRI study, the tracer first spreads to inferior regions of the parenchyma closer to the (modelled) injection site. Modelling a healthy patient, we assumed that the tracer concentration in the ventricular CSF was low [15, 16]. Thus, no tracer spreads to the parenchyma from the ventricles directly. In models with diffusion only, the amount of tracer in the gray matter peaks at approximately 15 h. In the MRI study, the time to peak enhancement in selected regions of interest was between 12 and 24 h [15]. In a more recent study, time to peak values were considerably longer, up to 48 h, for some regions [16]. However, in the latter study, the time to peak enhancement was shorter for the white matter than for the gray matter in healthy subjects. This observation is not consistent with the results from either of our computational models.

Most of the reported time to peak values in the two human MRI-studies [15, 16] are within the $99.73\%$ prediction interval of the random homogeneous diffusion model (Model D1). However, even for the upper range of the prediction interval, the time to peak/steady state value for the white matter exceeds 24 h in our model. The uncertainty in the diffusion coefficient may explain a fourfold difference in the amount of tracer found in the white matter at 24 h. Despite this large variation, the discrepancy between simulations and experiments in white matter could not be explained by uncertainty in the diffusion parameter. This may suggest other mechanisms in addition to diffusion for tracer transport into deeper regions of the brain. According to paraarterial influx theories in general and the glymphatic theory in particular, tracer flows rapidly along and into the parenchymal PVS [1] distributing tracer to the gray matter. Hence, one may expect diffusion models to underestimate the amount of tracer in gray matter at a given time. However, is worth noting that we do not observe such an underestimation in our diffusion model, when compared to the experimental values [15]. In contrast, we do observe a delayed distribution of tracer in white matter.

Brain tissue is known to be both anisotropic and heterogeneous [26, 70, 71]. We found the variation due to spatial heterogeneity in the diffusion coefficient to be low. As the correlation length was small compared to the size of the the gray and white matter, a lack to tracer concentration in one local region was balanced by enhancement in a different local region. In addition, we note that representing the diffusion coefficient as a random variable or a random field yields the same expected value. Tracer distribution to large brain regions can thus be well approximated using an average diffusion constant if the spatial heterogeneity is present on a shorter length scale.

In models with convection, given a homogenized velocity of average magnitude 0.17  µm/s, tracer distribution depends on the characteristics of the velocity field. In the glymphatic theory, CSF enters the brain along arteries and re-enters the SAS along a paravenous outflow pathway [1, 2]. In our glymphatic circulation model, the stochastic velocity field, representing homogenized paraarterial and paravenous flow, did not increase tracer distribution to the brain. An increase in the amount of tracer surrounding paraarterial spaces was balanced by a lower distribution around paravenous spaces. However, when local regions are addressed, tracer concentration may increase by up to 13% compared to diffusion alone, depending on the surrounding velocity field and region of interest. As we consider a homogenized representation of the PVS, this change reflects an increase in regions surrounding arterial PVS (not only inside the PVS). Iliff et al. [12] reported a twofold increase in tracer intensity in PVS in normal mice compared to mice with internal carotid artery ligation. The increase in the surrounding parenchyma was lower, approximately 30–40%, which compares more naturally with our estimate of 13%. It should be noted however, that our region of interest was deeper into the parenchyma (extending from 0.6 to 4 mm depth) than the region of interest (at 100  µm) used by Iliff et al. [12]. Moreover, our model parameters reflect a different species (man versus mouse), and the tracer spread takes place at a longer time scale.

When modelling paraarterial influx combined with parenchymal drainage (Model V3), the time to peak was reduced to 6–8 h in the gray matter. Although lacking quantitative drainage parameters, we observe that substantial clearance would reduce both the time to peak and relative tracer enhancement in the brain compared to diffusion alone. In the glymphatic directionality model (Model V2), guided by [59], the presence of a paravascular directional velocity also decreases the expected time to peak tracer enhancement in gray matter, down to 11 h (compared to 15 h for pure diffusion). Thus, when experimental data suggests a time to peak enhancement shorter than for diffusion alone, it is not clear whether this is due to increased glymphatic function or increased clearance by parenchymal drainage.

In our models, the white matter (and subregions) is where the effect of a convective velocity becomes most prominent. The only model modification causing an expected time to peak enhancement in white matter of approximately 24 h is with a paraarterial inflow and drainage (Model V3). In this model, the upper limit of the 99.73% prediction interval peaks at approximately 12 h, which is more comparable to the rapid tracer enhancement observed in the white matter of healthy subjects [16].

Although diffusion may act as the main transport mechanism in the parenchyma [9, 31], we here show that convective velocities of magnitude less than 1 µm/s may play an important role for transport. This result holds when there is a structure of the glymphatic circulation as used in Model V2 or possibly a net inflow as in Model V3. It should be noted that this directional velocity field, in which pulsations propagate upwards from the brain stem [58, 59], favors inflow when tracer is injected in lower CSF regions such as e.g. in the spinal canal.

### Limitations

In the present study, we have used a continuous and homogenized model of the brain parenchyma allowing only for an averaged representation of paravascular spaces on the scale of micrometers. To remedy this limitation, combined with restrictions placed by mesh resolution, we used lower velocities acting over larger areas to model paravascular flows. Clearly, the components of the brain parenchyma, including the vasculature, paravascular, extracellular and cellular spaces have dissimilar properties, and thus a homogenized model can only capture larger-scale features. At the same time, homogenized models are well-established for modelling fluid flow and transport in biological and geological porous media, see e.g. [72].

Further, we did not distinguish between white and gray matter in terms of the fluid velocity or in the diffusivity, although white matter is assumed to be more permeable [73]. However, in the absence of substantial drainage, net movement of fluid (in gray matter and PVS vs white matter) should on average be equal in the two regions by conservation of mass. Therefore, we used maximal velocity magnitudes of approximately 0.5  µm/s, which is similar to what has been reported in white matter [57], but not as high as has been reported in local regions in the PVS [13, 27]. While we used qualitative measurements [58, 59] to suggest a directionality in the glymphatic circulation, we predict that more detailed measurements of glymphatic function in different brain regions would be important for tracer enhancement and clearance.

The boundary concentration in our model was assumed to spread in a manner similar to what was seen from the signal intensity in the MRI study by Ringstad et al. [15]. A more detailed analysis of the spread of tracer in the CSF could be based on at least solving the Navier–Stokes equations in the SAS. In addition, our model ignores other efflux pathways directly from the SAS, such as e.g. arachnoid granulations [74], dural lymphatics [75, 76], and nasal lymphatics [77], although CSF drainage through the cribriform plate and other perineural routes eventually reaching the lymphatic system has recently been proposed to dominate glymphatic clearance [78]. By ignoring other efflux pathways over a time span of 24 h, we assume a relatively long terminal phase half-life of gadobutrol in the SAS. To the authors’ knowledge, this value is not well known. However, the data available suggest high concentrations of gadobutrol within the brain even after 24 h [15, 16], suggesting a half-life longer than our simulation time.

In the experiments by Ringstad et al. [15, 16], tracer distribution within the parenchyma varied considerably from patient to patient. In our analysis, we did not consider patient-specific meshes, but rather one representative mesh. Patient-specific meshes would add additional dimensions to the space of uncertainty, possibly giving different distributions in output in each of the patients.

The MRI-studies [15, 16] only provide quantitative values of tracer enhancement signal intensity, and not tracer concentrations. As the relation between signal intensity and concentration is nonlinear [79], we have not made a direct comparison between these two quantities. However, we have assumed that a peak in signal intensity corresponds to a peak in tracer concentration, thus allowing for a comparison of time-to-peak between the model results and experiments.

In our study, we assumed the probability distributions of the velocity and diffusivity coefficients to be known. In theory, it would be ideal to identify or learn these distributions from patients’ data via e.g. a Bayesian approach. Techniques for (infinite-dimensional) Bayesian inference [80, 81] have successfully been applied to fluid dynamics problems [82] and to brain imaging [83]. However, these methods require suitable quantitative data which are generally not available. In particular, we note that MRI only gives values of tracer enhancement signal intensity directly, and not tracer concentration or fluid velocities.

In this study, we considered a linear reaction–convection–diffusion equation as a standard and classical model for the evolution of a solute concentration. At the same time, we introduced a set of modelling assumptions for the velocity and diffusivity fields. An alternative approach could be to identify the mathematical model via inverse modelling, model adaptivity or learning based approaches. Given suitable data and a set of feasible models, it could be possible to identify or learn the models and/or model parameters that best represent the in vivo observations.

## Conclusions

The results from this study show that uncertainty in the diffusion parameters substantially impact the amount of tracer in gray and white matter, and the average tracer concentration in gray and white subregions. However, even with an uncertainty in the diffusion coefficient of a factor three, and a resulting fourfold variation in white matter tracer enhancement, discrepancies between simulations of diffusion and experimental data are too large to be attributed to uncertainties in the diffusion coefficient alone.

A convective velocity field modelling the glymphatic theory, with arterioles and venules placed at random, did not increase tracer enhancement in the brain parenchyma compared to pure diffusion. However, when a large-scale directional structure was added to this glymphatic velocity field, tracer inflow increased.

Diffusion alone was able to mimic behaviour in MR-studies in specific regions. However, this result does not imply lack of glymphatic circulation as the gray matter tracer enhancement was equal for the glymphatic model with directionality and for diffusion alone. On the other hand, the white matter concentration was greatly increased in the former model. Thus measuring glymphatic function requires detailed experimental data and analysis of the whole brain.

## Supplementary information

**Additional file 1.** Uncertainty quantification of parenchymal tracer distribution using random diffusion and convective velocity fields consisting of Sections A, B, C. Section A includes additional descriptions of the stochastic field modelling. Section B describes the numerical solution of the convection-diffusion-reaction equations. Section C presents the model verification.

## Data Availability

The datasets generated and analyzed during the current study are available via the Uncertainty quantification of parenchymal tracer distribution using random diffusion and convective velocity fields (data sets): https://doi.org/10.5281/zenodo.3241364. Additional data and computer code are available from the corresponding author on reasonable request.

## Abbreviations

CDF: cumulative density function
CSF: cerebrospinal fluid
ISF: interstitial fluid
MR(I): magnetic resonance (imaging)
MC: Monte Carlo
PDE: partial differential equation
PDF: probability density function
PVS: paravascular/perivascular space(s)
SAS: subarachnoid space
UQ: uncertainty quantification

## Footnotes

1 A stochastic or random model is a model incorporating one or more elements of uncertainty or randomness. The opposite of stochastic is referred to as non-stochastic, non-random or deterministic.
