# Supplementary material for: Uncertainty quantification of parenchymal tracer distribution using random diffusion and convective velocity fields
Source: Fluids Barriers CNS. 2019 Sep 30;16:32. doi: 10.1186/s12987-019-0152-7 (PMC6767654; doi:10.1186/s12987-019-0152-7)
Supplement: Supplementary file 1 — Additional file 1. Uncertainty quantification of parenchymal tracer distribution using random diffusion and convective velocity fields consisting of Sections A, B, C. Section A includes additional descriptions of the stochastic field modelling. Section B describes the numerical solution of the convection-diffusion-reaction equations. Section C presents the model verification. [file 12987_2019_152_MOESM1_ESM.pdf]

Uncertainty quantification of parenchymal tracer distribution using random diffusion and convective velocity fields

M. Croci, V. Vinje and M. E. Rognes

A Stochastic field modelling

A.1 Gaussian and Matérn fields

Let Y be a random field over a spatial domain $\mathcal{D} \subseteq \mathcal{R}^3$. In the specific case in which all the random variables that form the random field are Gaussian random variables, then the field is called a *Gaussian field* [1]. A Gaussian field, similarly as a Gaussian random variable, only depends on two parameters: the mean $\mu(x)$ and the covariance function $\mathcal{C}(x, y)$ (the probability density of a Gaussian random variable is uniquely determined by mean and variance).

A Gaussian field is also a Matérn field when its covariance is a Matérn covariance, i.e. of the form

$$\mathcal{C}(x, y) = \frac{\sigma^2}{2^{\nu-1}\Gamma(\nu)}(\kappa r)^{\nu}\mathcal{K}_{\nu}(\kappa r) \tag{1}$$

for $x, y \in \mathcal{D}$, where r is the distance between x and y: $r = \|x - y\|$, $\kappa = \frac{\sqrt{8\nu}}{\lambda}$, $\Gamma(x)$ is the Euler Gamma function, and σ^2, ν, $\lambda > 0$ are the variance, smoothness parameter and correlation length of the field respectively and $\mathcal{K}_{\nu}$ is the modified Bessel function of the second kind.

Matérn fields are extensively used in spatial statistics, biology and oil reservoir modelling to represent uncertain or randomly-varying fields [2, 3]. The smoothness parameter ν regulates the field's spatial smoothness: field samples are almost surely continuous and $\lceil \nu \rceil - 1$ times differentiable [1]. For the two cases $\nu = 1/2$ and $\nu = \infty$, (1) reduces to the exponential and Gaussian covariance functions, respectively. The correlation length λ roughly represents the distance past which point values of the field are approximately uncorrelated. Informally, this means that in each realization of the Matérn field, there are regions of length proportional to λ within which the values of the field are similar.

A.2 Random field representation of the effective diffusion coefficient

To define the random effective diffusion field (Model D2), we let

$$D^*(x, \omega) = 0.25\, D^*_{\text{Gad}} + D^*_f(x, \omega), \tag{2}$$

where D^*_f is a random field such that for each fixed $x \in \mathcal{D}$, $D^*_f(x, \cdot)$ is a gamma-distributed random variable with shape $k = 3$ and scale $\theta = 0.75 \times D^*_{\text{Gad}}/k$. To enforce continuity and to easily sample the random field from its distribution, we draw samples of D^*_γ by first sampling a Matérn field $X(x, \omega)$ and then transforming it into a gamma random field by using a copula [4]. This consists in setting $D^*_\gamma(x, \omega) = F^{-1}(\Phi(X(x, \omega)))$, where F^{-1} is the inverse cumulative density function (CDF) of the target (gamma) distribution, Φ is the CDF of the standard normal distribution and $X(x, \omega)$ is a standard (zero mean, unit variance) Matérn field with smoothness parameter $\nu = 2.5$ and correlation length $\lambda = 0.01$ m, cf. (1). Note that spatial changes in the diffusivity occurs at a length scale corresponding to the correlation length, here 0.01 m.

Species	Brain mass [g]		d_{CCA} [mm]		d_{A} [μm]		Δ_{AV} [μm]	
Mouse	0.3	[8]	0.47	[9]	25	[10]	40*	
Monkey	88	[8]	3.5	[11]	35.5	[5]	280	[5]
Human	1350	[8]	6.3	[12]	40-250	[13]	1020*	

Table 1: Brain-related parameters of three species. *: Estimated values. d_{CCA}: diameter of the common carotid artery, d_{A}: arteriole diameter Δ_{AV}: distance between arteriole and venule.

A.3 Random field representation of the glymphatic velocity model

We define the stochastic glymphatic circulation velocity field (Model V1) by

$$v(x,\omega) = v_{\mathrm{avg}} \cdot \eta(\lambda)\ 10^{-\mathcal{E}(\omega)} \left(\nabla \times \begin{bmatrix} X(x,\omega) \\ Y(x,\omega) \\ Z(x,\omega) \end{bmatrix} \right), \tag{3}$$

where $\eta(\lambda) = \lambda/\sqrt{(5+2\log(10))/200}$ is a scaling constant chosen such that the magnitude of v satisfies $\mathbb{E}[||v||^2]^{1/2} = v_{\mathrm{avg}}$ (we omit the mathematical derivation of this constant), $\mathcal{E}(\omega)$ is an exponentially distributed random variable with mean 0.2 and $X(x,\omega)$, $Y(x,\omega)$ and $Z(x,\omega)$ are standard independent identically distributed (i.i.d) Matérn fields with $\nu = 2.5$ and correlation length $\lambda = 1020\,\mu$m.

The factor $10^{-\mathcal{E}(\omega)}$ is an ad-hoc random term to enforce the variability requirement. The use of Matérn fields enforces spatial variability in a continuous manner and taking the curl operator ($\nabla\times$) ensures that the resulting velocity is divergence-free. It can be proven (although we omit the details here) that the field within the brackets in (3) is still Gaussian, has zero mean and has the same correlation length as the original Matérn fields, albeit it presents a slightly different covariance structure.

The choice of correlation length was guided by the following considerations. The mean distance between arterioles and venules was reported to be 280 μm in rhesus monkeys [5], although the value 250 μm has been used as a representative distance in humans in recent modeling papers [6, 7]. We estimated the mean distance in humans by considering differences in brain and artery size between monkey and human (Table 1). We find a factor close to 2 between CCA and arteriole diameter, while a similar ratio was found for the cube root of the brain mass. Thus, the correlation length should be greater than $250 - 560\mu$ m. Combining these physiological considerations with the corresponding requirements on the numerical resolution, we let $\lambda = 1020\,\mu$m.

B Numerical solution of the convection-diffusion-reaction equation

Overall, the diffusion-convection equation was solved numerically using a finite element method with continuous piecewise linear finite elements in space, and an implicit midpoint finite difference discretization time with time step $\Delta t = 15$ min, combined with mass lumping [14]. We detail these steps in the following.

Time discretization After time discretisation of the PDE, we obtain for each time step n: given c^n, find c^{n+1} such that

$$\frac{c^{n+1} - c^n}{\Delta t} + \frac{1}{2}(F(c^{n+1}) + F(c^n)) = 0, \tag{4}$$

with $c^0 = 0$ and where

$$F(c) = \nabla \cdot (vc) - \nabla \cdot (D^* \nabla c) + rc. \tag{5}$$

We approximate the Dirichlet boundary condition explicitly and by using a trapezoidal rule for the time integration, i.e we let

$$c^{n+1}(x) = c_{\mathrm{CSF}}^{n+1}\, h(t^{n+1}, x) \quad \text{for } x \in \partial\mathcal{D}_S, \tag{6}$$

where

$$c_{\mathrm{CSF}}(t^{n+1}) \approx c_{\mathrm{CSF}}^{n+1} = \frac{1}{V_{\mathrm{CSF}}}\left(n_0 - \int_{\mathcal{D}} c^n \,\mathrm{d}x - \frac{\Delta t}{2}\left(2\sum_{i=1}^{n-1}\int_{\mathcal{D}} rc^i \,\mathrm{d}x + \int_{\mathcal{D}} rc^n \,\mathrm{d}x\right)\right). \tag{7}$$

Here the term in the inner bracket results from the numerical integration of the term $\int_0^{t_n}\int_D rc \,\mathrm{d}x\,\mathrm{d}t$. The explicit (first-order) discretization of the boundary term reduces the overall accuracy of the scheme to first-order in time.

Spatial discretization We discretize (4) in space using the finite element method. Given a finite element space $V_h \subseteq H^1(\mathcal{D})$ and $V_h^\circ \subset V_h$ such that $s = 0$ on $\partial\mathcal{D}_S$ for all $s \in V_h^\circ$, the fully discrete scheme reads as follows. Let $c_h^0 = 0$. For each time step n, given $c_h^n \in V_h$, find $c_h^{n+1} \in V_h$ such that, for all test functions $s_h \in V_h^\circ$,

$$\int_{\mathcal{D}} \frac{c_h^{n+1} - c_h^n}{\Delta t} s_h \,\mathrm{d}x + \frac{1}{2}\left(a(c_h^{n+1}, s_h) + a(c_h^n, s_h)\right) = 0, \tag{8}$$

where

$$a(c, s) = \int_{\mathcal{D}} \nabla\cdot(vc)s + D^*\nabla c\cdot\nabla s + rc\,s \,\mathrm{d}x, \tag{9}$$

and such that

$$c_h^{n+1} = c_{\mathrm{CSF}}^{n+1,h} h(t^{n+1}, x) \quad \text{for } x \in \partial\mathcal{D}_S \tag{10}$$

and $c_{\mathrm{CSF}}^{n+1,h}$ is given by (7) in which c^n and c^i are replaced by c_h^n and c_h^i respectively. We choose the FEM subspaces to be spanned by continuous piecewise linear basis functions.

Péclet number We remark that the problem is mildly convection-dominated, with an upper estimate of the Péclet number of

$$Pe \approx \frac{9Lv_{\mathrm{avg}}}{D_{\mathrm{Gad}}^*} \approx O(10^3), \tag{11}$$

where $L \approx 0.084$ m is half the diameter of the computational domain, $v_{\mathrm{avg}} = 0.17\mu$m/s, and $D_{\mathrm{Gad}}^* = 1.2 \times 10^{-10}$ m/s^2. The factor 9 was added to make larger values extremely unlikely. Given the mesh sizes in question, we obtain low-probability worst-case cell Péclet numbers of $\approx O(10)$ for the mesh used for the simulations. In numerical experiments, numerical instabilities due to convection were not observed. We thus did not use stabilization techniques for the main numerical simulations.

C Model verification

To test the numerical approximations with respect to choice of mesh and time-step parameters, we performed convergence tests for all output quantities of interest. We tested three different meshes resulting from adaptive and uniform refinement, and three different time steps. The original mesh [15] was refined locally close to the outer boundary, as refinement was needed in these regions of steep concentration gradients in the first time steps. We also tested that the SUPG-method performed similar to the other methods and confirmed that the SUPG-method was not needed for stability on finer meshes. The results are shown in Figures 1 and 2.

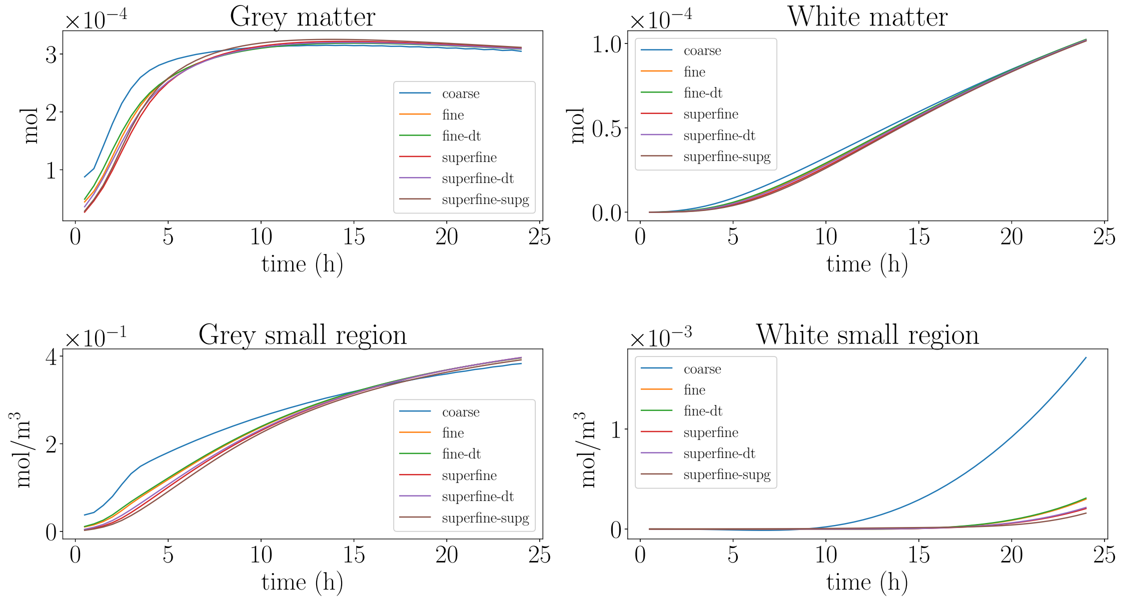

Figure 1: Convergence plots of Model V2 for a given deterministic velocity magnitude used to set up the velocity field. Due to high velocities, the mesh needs refinement to avoid considerable overshoot in the small white matter region.

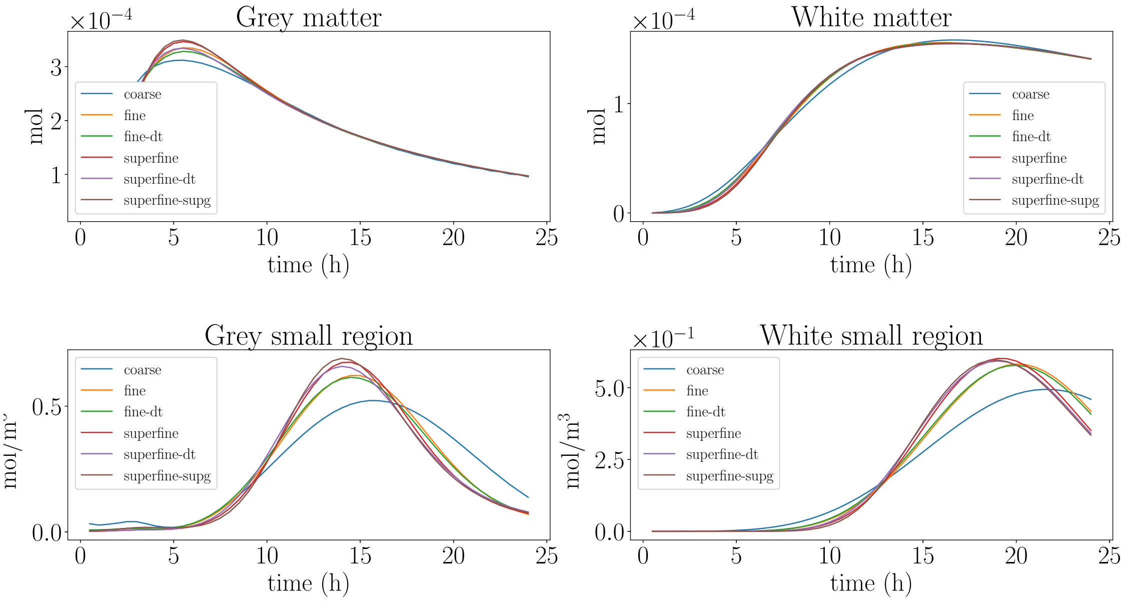

Figure 2: Convergence plots of Model V3 for a given deterministic velocity magnitude used to set up the velocity field. The local regions are more prone to error than the gray and white matter as a whole.

References

[1] Abrahamsen P. A Review of Gaussian Random Fields and Correlation Functions. 2nd ed. Norwegian Computing Center; 1997.

[2] Potsepaev R, Farmer CL. Application of stochastic partial differential equations to reservoir property modelling. In: ECMOR XII-12th European Conference on the Mathematics of Oil Recovery. vol. 2; 2014. .

[3] Lindgren F, Rue H, Lindström J. An explicit link between Gaussian fields and Gaussian Markov random fields: the stochastic partial differential equation approach. Journal of the Royal Statistical Society: Series B (Statistical Methodology). 2009;73(4):423–498.

[4] Nelsen RB. An introduction to copulas. Springer Science & Business Media; 2007.

[5] Adams DL, Piserchia V, Economides JR, Horton JC. Vascular supply of the cerebral cortex is specialized for cell layers but not columns. Cerebral Cortex. 2015;25(10):3673–3681.

[6] Jin BJ, Smith AJ, Verkman AS. Spatial model of convective solute transport in brain extracellular space does not support a 'glymphatic' mechanism. The Journal of general physiology. 2016;148(6):489–501.

[7] Ray L, Iliff JJ, Heys JJ. Analysis of convective and diffusive transport in the brain interstitium. Fluids and Barriers of the CNS. 2019;16(1):6.

[8] Roth G, Dicke U. Evolution of the brain and intelligence. Trends in cognitive sciences. 2005;9(5):250–257.

[9] Lacolley P, Challande P, Boumaza S, Cohuet G, Laurent S, Boutouyrie P, et al. Mechanical properties and structure of carotid arteries in mice lacking desmin. Cardiovascular research. 2001;51(1):178–187.

[10] Iliff JJ, Wang M, Zeppenfeld DM, Venkataraman A, Plog BA, Liao Y, et al. Cerebral arterial pulsation drives paravascular CSF–interstitial fluid exchange in the murine brain. Journal of Neuroscience. 2013;33(46):18190–18199.

[11] Ye Z, Liu Y, Wang X, Chen X, Lin C, Su Y, et al. A rhesus monkey model of common carotid stenosis. Int J Clin Exp Med. 2016;9(9):17487–17497.

[12] Krejza J, Arkuszewski M, Kasner SE, Weigele J, Ustymowicz A, Hurst RW, et al. Carotid artery diameter in men and women and the relation to body and neck size. Stroke. 2006;37(4):1103–1105.

[13] Auer RN. Histopathology of Brain Tissue Response to Stroke and Injury. In: Stroke (Sixth Edition). Elsevier; 2016. p. 47–59.

[14] Thomée V. On positivity preservation in some finite element methods for the heat equation. In: International Conference on Numerical Methods and Applications. Springer; 2014. p. 13–24.

[15] Fang Q. Mesh-based Monte Carlo method using fast ray-tracing in Plücker coordinates. Biomedical optics express. 2010;1(1):165–175.
